# Sustainable Return to Work: A Systematic Review Focusing on Personal and Social Factors

**DOI:** 10.1007/s10926-019-09832-7

**Published:** 2019-02-15

**Authors:** Abasiama Etuknwa, Kevin Daniels, Constanze Eib

**Affiliations:** 1 Norwich Business School, University of East Anglia, Norwich Research Park, Norwich, NR47TJ UK; 2 Department of Psychology, Uppsala Universitet, Von Kraemers allé 1A och 1C 752 37, Box 1225, 751 42 Uppsala, Sweden

**Keywords:** Return to work, Musculoskeletal pain, Mental disorders, Systematic review, Occupational health

## Abstract

*Purpose* A systematic review was conducted to evaluate the impact of important personal and social factors on sustainable return to work (RTW) after ill-health due musculoskeletal disorders (MSDs) and common mental disorders (CMDs) and to compare the effects of these personal and social factors across both conditions. Sustainable RTW is defined as a stable full-time or part-time RTW to either original or modified job for a period of at least 3 months without relapse or sickness absence re-occurrence. *Methods* A literature search was conducted in 13 databases and 79 studies were selected for the review, of which the methodological design was graded as very high, high and low quality. *Results* The most consistent evidence for achieving sustainable RTW for both MSDs and CMDs was from support from line managers or supervisors and co-workers, positive attitude, self-efficacy, young age and higher education levels. Job crafting, economic status, length of absence and job contract/security showed promising results, but too few studies exist to draw definite conclusions. Results regarding gender were inconsistent. *Conclusions* This review demonstrates that a variety of personal and social factors have positive and negative influences on sustainable RTW. We suggest that the social environment and how it interrelates with personal factors like attitudes and self-efficacy should be studied in more detail in the future as the inter-relationship between these factors appears to impact positively on sustainable RTW outcomes. Areas for future research include more high-quality studies on job crafting, economic status/income, length of absence, job contract/security and gender.

**Electronic supplementary material:**

The online version of this article (10.1007/s10926-019-09832-7) contains supplementary material, which is available to authorized users.

## Introduction

Musculoskeletal and common mental disorders (MSDs and CMDs) have been recognized as the most common causes of sickness absence in developed countries, and it has become a major research focus, especially as the economic cost on sickness absence is growing yearly [1]. In 2014/15, approximately 1.2 million workers in Great Britain were suffering from ill-health that was either caused or worsened by their current or past jobs [2]. Of the 1.2 million workers, 80% of work-related illness was due to musculoskeletal disorders (MSDs) and common mental health disorders (CMDs) such as stress, depression or anxiety [2]. These figures constitute significant fractions of reported sickness absence episodes, and extended absence is associated with reduced probability of return to work (RTW) [3], which becomes costly for employers, increasing the urgency to help workers RTW early.

To reduce costs related to sickness absence and reduce the risk of long-term disability associated with extended absence from work, there is a big need for a better understanding of the factors that either impede or facilitate a sustainable RTW for workers sick-listed with MSDs and CMDs. Although studies have shown how work can instigate ill-health such as MSDs and CMDs [4, 5], there is also strong evidence that work is an important component for a speedy recovery after ill-health episodes and that work is generally beneficial for physical and mental health [6, 7].

Until now, systematic reviews on RTW have to a great extent focused on the effectiveness of a varied number of interventions [8–17]. However, it is still unclear what factors are effective in facilitating sustainable RTW outcomes [18, 19]. We defined sustainable RTW as a stable full-time or part-time RTW to either original or modified job for a period of at least 3 months without relapse or sickness absence re-occurrence. According to Cancelliere et al.’s [18], the process of RTW is complex and not merely dependent on the effectiveness of interventions, rather it involves an interplay of many factors beyond the health condition. Similarly, Alavi and Oxley [6] assert that when research concentrates more on learning about factors associated with sustainable RTW, further gains will be achieved in the effectiveness of RTW programmes.

Cancelliere et al. [18] conducted a systematic review of reviews to identify prognostic factors for RTW and their association with RTW outcomes. Cancelliere et al.’s study [18] identified higher education levels, higher socio-economic status, higher self-efficacy and optimistic expectations for recovery and RTW, lower severity of injury/illness, better RTW coordination and multidisciplinary interventions as common prognostic factors associated with a positive RTW. Cancelliere et al.’s [18] findings introduced a promising line of direction; that employee’s personal and social relations in the workplace both play an important role for better understanding RTW. However, sustainable RTW was not the outcome measure in that review, and ill-health was not limited to MSDs and CMDs but extended across different health and injury conditions. Thus, there warrants a review specifically addressing sustainable RTW outcomes for people with MSDs and CMDs. Similarly, Gallagher et al. [20] suggested that lasting RTW outcomes may be achieved through employees’ personal factors like age and length of sickness absence and psychosocial factors like social support, health locus of control and illness behaviour. In recent times, there has been similar suggestions to improve RTW models and policies to take into account these personal and social factors in the workplace [21–24]. However, there are currently no reviews explicitly investigating the effects of personal and social factors on sustainable RTW outcomes for MSDs and CMDs, as such, a review like our current review could help uncover the factors that can account for the stability of absence due to MSDs and CMDs in advanced economies, in spite of evidence for the effectiveness of RTW interventions [8–11]. Additionally, in the current literature on RTW, there is a heavy focus on MSDs, especially low back pain and little on CMDs [18]. This review seeks to address these gaps in evidence, thus providing a unique contribution to the literature on sustainable RTW after ill-health due to MSDs and CMDs.

Very few guidelines on sickness absence management address both MSDs and CMDs holistically, although there are striking parallels between both conditions [25]. Both conditions share similarities in health characteristics relating to delayed onset, delayed recovery, reduced life expectancy and unclear diagnosis which in many cases may result in chronic absences [26, 27]. The RTW processes and psychosocial risk factors for these conditions are also similar [26, 27]. According to Heuvel [28], even though psychosocial risk factors are often associated with CMDs, several studies have demonstrated that they also have an effect on MSDs. The association between MSDs and CMDs has been widely investigated, and findings indicate that people of working age with CMDs are often coexisting with MSDs which may influence a person’s successful RTW [29]. Therefore, there are several reasons to investigate RTW outcomes for both MSDs and CMDs together.

This review focused on identifying various employee’s personal and social factors taken into account in both intervention and non-intervention-based studies reporting sustainable RTW outcomes for people sick-listed with MSDs and CMDs. Sustainable RTW is difficult to define especially as different studies use varying durations for outcome measures because of the difference in absence duration for MSDs and CMDs [30]. According to Krause et al. [31], because measures of duration of disability and RTW outcomes serve multiple functions in principle, it becomes important to clearly state the function of outcome measures. As such the function of sustainable RTW outcome in this review was to identify a stable period of return after sick-leave without a relapse. Jensen et al. [32] defined sustainable RTW for people sick-listed with MSDs as the first period of four consecutive weeks without receiving health-related benefits. They argued that the 4-week period without relapse was considered sufficiently long enough to suggest a lasting and stable return. Conversely, Lammerts et al.’s [10] study on sick-listed workers with a depressive or anxiety disorder operationalized sustainable RTW as employed participants who have not been long-term sick-listed (more than 14 days) in the previous 6 months. Hoefsmit et al. [22] investigated RTW outcomes for employees sick-listed with all ill-health apart from terminally ill employees, and defined sustainable RTW as working for four weeks without relapse in partial or complete sick-leave. In this review, sustainable RTW was formulated with a timeframe of at-least 3 months without relapse or absence. Across the included studies in this review, 3 months was the lowest follow-up period of which successful return to full-time and part-time work was recorded for people sick-listed with both MSDs and CMDs. Like Jensen et al. [32], we argue that RTW for at-least 3 months with no recorded incidence of relapse and subsequent absence is considered a sufficiently long enough timeframe to suggest sustainability of return for people with both conditions. The 3 months’ timeframe also takes into account the different recovery and RTW period for both MSDs and CMDs identified in previous studies.

The aim of this systematic review was to assess the impact of personal and social factors on a sustainable RTW after ill-health due to MSDs or CMDs. In addition, we aimed to identify commonalities of effects of these personal and social factors between both conditions. Personal factors identified included attitude, self-efficacy, age, gender, education, economic status/income, length of sickness absence, job contract/ security. Social factors identified included support from leaders and co-workers (where leaders include managers, line managers, supervisors etc.) and job crafting and its related practices (employee-initiated changes to job or how work is done). Job crafting refers to employees redesigning their job task to fit their motives, strengths and passions [33, 34]. This concept of job redesign helps to capture the actions employees independently take to shape, mould and redesign their jobs [35]. According to Wrzesniewski and Dutton [35], by crafting one’s job, individuals are accorded the opportunity to change not just the elements of their jobs, but also their relationship with others to redefine the meaning of their work and the social environment of their work.

Findings from this review will help us understand what factors may either instigate or hinder a sustainable RTW. The review intends to provide employers and policy makers with knowledge of key factors that will aid in implementing more effective RTW programmes. It will also add to the body of evidence on the impact of personal and social factors on RTW outcomes which is currently limited [16], inform policy decision making and provide avenues for future research in the field of RTW.

## Methods

The systematic review was conducted in line with the Preferred Reporting Items for Systematic Reviews and Meta-Analyses (PRISMA) guidelines [36]. The protocol was duly developed prior to the review and registered with PROSPERO (https://www.crd.york.ac.uk/PROSPERO/display_record.asp?ID=CRD42016053967) (registration no; CRD42016053967).

### Literature Search

A systematic review of sustainable RTW after ill health was conducted. A search strategy based on PICOS strategy was formulated [37]. This strategy allows its five components (population, intervention, comparator, outcome and study design) to be taken into account when developing a search strategy that is unbiased, reproducible and helps to rapidly and accurately locate the best available and relevant scientific literature that fit into the scope of the review and answer the research questions [38].

However, because this review had no specific comparator, the research question was derived in terms of the participant, intervention, outcome related to the risk posed and study design suitable for addressing it (PIOS) [37, 39].The search inclusion criteria included studies that reported on employees returning to work after absence due to MSDs or CMDs (*population*), the effects of personal and social factors on RTW outcomes (*intervention*), a sustained RTW after ill-health such as MSDs or CMDs (*outcome*) and studies of all designs published in English from 1989 to 2017. Out of a need to accurately assess RTW approaches and interventions that have taken into account personal and social factors, the timeframe was extended to include 1989. Even though research as far back as 1989 may not necessarily provide evidence generalizable in today’s work environment, it was considered relevant to include this research because this range included an early, if not the earliest paper that explored the association between multiple personal and social factors and successful RTW [20]. Based on this, search terms were developed, and the first author conducted a comprehensive search of relevant electronic databases including published and unpublished research, grey literature and reference lists of both primary studies and reviews. Table 1 shows the search terms that the first author adopted during the search. The search was conducted between October 2016 and March 2017 on 13 databases: Business Source Complete, CINAHL, Cochrane Library, EBOSCO Host, JSTOR, Medline (OVID), Psych INFO, PubMed, Scopus, ScienceDirect, SPORT Discus, Web of Science and Wiley Online Library (see Online Resource 1 for a summary of the search result for each database).

**Table 1:** Search terms used

	Population	Intervention	Outcome	Study design
Possible search terms	• Return* to work employee*	• Leader*	• Sustain* return* to work	• Randomi*controlled trial*
	• Return* to work officer*	• Co-workers	• Bearable return* to work	• Intervention*
	• Return* to work worker*	• Social support	• Endurable return* to work	• Cohort
	• RTW rehab*	• Employee* character	• Sustain* recovery	• Experimental
	• Occ* rehab*	• Job crafting	• Back to work	• Randomi*
	• Employee*	• Managers	• Sustain* back to work	• Trial*
	• Absent from work	• Supervisors	• Bearable back to work	• ‘Clinical Trial’ [publication type]
	• Worker* absence from work	• Colleagues	• Endurable back to work	• “Meta-analysis” [publication type]
	• Return* to work staff	• Job re-design	• Workability	• Quasi-experiment
	• Employee* returning from ill-health	• Job altering		• Systematic review
	• Worker* returning from ill-health	• Organi* changes		• Evidence synthesis
	• Staff returning from ill-health	• Personal trait		• Observational
	• Employee* with MSDs	• Individual difference		• Qualitative
	• Worker* with MSD	• Supervision		• Survey
	• Staff with MSDs	• Adaptation*		• Mixed
	• Employee* with depression	• Interventions		• Quantitative
	• Worker* with depression	• Job modification		
	• Staff with depression	• Climate		
	• Sickness presence	• Vocational		
	• MSDs	• Rehab*		
	• Musculoskeletal disorders	• Supported employment		
	• Depression	• Work adjustment		
	• Mental health issues	• Occupation* adjustment		
	• Ill-health	• Workplace intervention		
	• Time loss from work	• Modified work		
		• Occupational intervention		

### Selection of Studies

The first author conducted the selection of relevant studies in three stages: (i) Title; (ii) Abstract; and (iii) Full-Text/Paper screening. A title screening was conducted to retrieve papers specifically reporting RTW outcomes for CMDs and MSDs. At this stage, if the study indicated the RTW outcome for ill-health other than MSDs and CMDs, the article was excluded. Identified citations were further sifted according to the abstract, to select citations eligible for possible inclusion in the review.

In the third stage, the first author assessed the full-text/paper for quality and relevance to the research question. Where a study did not meet the inclusion criteria, the paper was excluded. All retrieved studies were screened independently by the first author and 30% each further checked by the other authors to ensure reliability and transparency in the selection process, consistency in interpretation and eligibility of included studies in the final review.

### Quality Appraisal

Methodological quality of individual studies was assessed using the Critical Appraisal Skill Programme (CASP) Checklist for qualitative and mixed studies and the checklist of evidence quality adapted from the “Early Intervention Foundation” (EIF) for quantitative studies adapted from Snape et al. [40]. Each aspect of the study was given a quality rating (‘yes’, ‘no’ or ‘can’t tell)’ based on the criteria on the checklist [40] (see Online Resources 8 and 9 for assessment tools). Based on the checklist criteria, studies were considered of good methodological quality and therefore included in the review if the answers to all the screening question were ‘yes’. However, a concession was agreed also include studies that recorded a few ‘no’ or ‘can’t tell’ answers based the degree to which an evaluated factor has been shown to have a positive impact on specific outcomes (EIF) and on the relevance of findings, appropriate methodology and rigor in analysis (CASP). As a result, all studies were included in the summary regardless of the methodological quality. The first author independently assessed the methodological quality of each study using both assessment tools, of which the other authors checked for consistency to address inter-rater reliability.

The final quality grading for the quantitative studies was based on the grading recommendations assessment development and evaluation (GRADE) approach [41], the qualitative and mixed studies were based on the confidence of evidence from reviews of qualitative research (CERqual) [42]. In GRADE, multiple randomized controlled trials (RCTs) with good statistical power converging on reliable effect sizes with narrow intervals are considered as ‘high-quality’ evidence. Well-designed observational studies with good statistical power are considered as ‘low-quality’ evidence. However, GRADE allows flexibility in rating evidence at a higher or lower level depending on a range of considerations. For example, evidence initially rated as ‘high-quality’ can be downgraded due to study limitations, inconsistency of results, indirectness of evidence, imprecision and reporting bias. Similarly, evidence initially rated a ‘low-quality’ can be upgraded to high-quality if there is a very large magnitude of effect, a dose–response gradient, and all plausible biases would reduce an apparent treatment effect [40]. In this review RCTs were categorized as very high-quality and upgraded observational studies were categorized as high-quality to aid clear distinction between both study designs. CERqual approach uses a similar approach to the GRADE tool to grade the quality of evidence [40]. Qualitative and mixed studies were thus graded very high-quality based on four components. The methodological limitations of the studies contributing to a review finding, relevance to the review question of the studies contributing to a review finding, coherence of the review finding, and adequacy of data supporting a review finding.

Therefore, both GRADE and CERqual approaches were used to inform a final assessment of the quality of the findings of the review, as such, data extraction and evidence synthesis were completed on very-high, high and low-quality studies.

### Data Extraction

A data extraction form was designed using the PIOS (Population, Intervention, Outcome and Study Design) strategy to minimize the possible errors or biases that may occur at this stage [37]. This data extraction form was designed based on how the research question was formulated with a view to obtaining all the relevant information from included studies [43]. This strategy was helpful in gaining a deeper understanding of the evidence to prevent error in interpretation as well as enhanced transparency of the method of analysis [43]. Data extraction sheets were thus designed to capture all the necessary study details e.g. author, study design and more detailed information about the nature of the intervention, personal and social factors and the outcomes. To ensure consistent extraction of necessary information from the studies, the authors conducted a pilot exercise. Data were extracted from ten random papers by all of the authors, who then discussed any discrepancies or differences in interpretation of the papers to ensure consistent data extraction from all of the included articles. Following the pilot exercise, the data extraction sheet was augmented to require more information on papers to aid easy understanding and prevent returning to the original paper for clarification (see Online Resource 2 for the full data extraction sheet).

### Evidence Synthesis

Once data were extracted, the first author synthesized the data extraction sheets into an evidence summary table (See Online Resource 3). Since the outcome measures of included studies were very heterogeneous, data was synthesized using narrative synthesis. Hence a series of harvest plots (adapted from [44]) (see Online Resources 4, 5, 6, 7) and evidence statements summarizing the quality of evidence (see Table 2) were developed by the first author based on two distinct categories of ill-health (MSDs and CMDs). These plots are an effective means in visualizing findings in a way that takes the quality of study into account [45]. Each plot consists of three columns representing the three-competing hypotheses (positive effect, negative effect and no effect) and a bar represents each study in each of the columns according to the competing hypothesis results of the study supported. The row represents the domains of the evaluated personal and social factors (support from leaders, support from co-workers, job-crafting and personal characteristics). Based on the included studies, personal characteristics included positive attitude to work and the return to work process, high self-efficacy, younger age, gender, high education, low economic status/income, short-term length of absence and temporary or insecure job contract. The quality of evidence in the review is indicated by the height of the bar with a specific designation on it in each row (H to represent very high-quality studies, U to represent low-quality studies upgraded to high quality based on the GRADE criteria and L to represent low-quality studies, see below). Studies with relatively stronger designs (RCT) are indicated with full-tone (black) bars, and weaker study designs (observational and qualitative/mixed studies) are marked with half tone (grey) bars.

**Table 2:** Summary evidence statements with GRADE and CERqual ratings

Evidence statement (outcomes)	Rating	Reasoning
Support from leaders plays a role in facilitating sustainable RTW for employees with musculoskeletal disorders (MSDs)	Strong confidence (high level of evidence)	Seven randomized controlled trials were included, one of which was graded low quality as a result of a high risk of bias. Ten High-quality qualitative studies and one high-quality mixed study based on the CERqual criteria was included. Twenty-three observational studies initially rated low quality using the GRADE system were included. Nineteen of which were upgraded to high-quality studies using the GRADE upgrade criteria and four of which were graded low quality. As such, good quality studies were predominantly evaluated in this study
Support from co-workers plays a role in facilitating sustainable RTW for employees with musculoskeletal disorders (MSDs)	Strong confidence (high level of evidence)	Five high-quality qualitative studies and one high-quality mixed study based on the CERqual criteria were included. Eleven observational studies initially rated low quality using the GRADE system were included. Nine of which were upgraded to high-quality studies using the GRADE upgrade criteria and two of which were graded low quality. Although there were no randomized control trials, fifteen out of the seventeen included studies showed consistent positive effects on sustainable RTW
Job-crafting plays a role in facilitating sustainable RTW for employees with musculoskeletal disorders (MSDs)	Low confidence (low level of evidence)	Only three studies (one high quality randomized control trial, one high quality qualitative study and one observational study upgraded to high quality using the GRADE criteria) with consistent effects across all studies were included. Considering the small number of studies, more studies in the area will need to be conducted to produce strong conclusions on its effects
Personal characteristics play a role in facilitating sustainable RTW for employees with musculoskeletal disorders (MSDs)		
Attitude	Strong confidence (high level of evidence)	One very high-quality RCT and Two high-quality qualitative/ mixed based on the CERqual criteria were included. Eleven observational studies initially rated low quality using the GRADE system were included. Nine of which were upgraded to high-quality studies using the GRADE upgrade criteria and Two of which were graded low quality. Although there was only one randomized control trial, all sixteen included studies showed consistent positive effects on sustainable RTW
Self-efficacy	Moderate confidence (moderate level of evidence)	Four observational studies upgraded to high quality studies using the GRADE criteria were included. All studies showed consistent positive effect on sustainable RTW. Regardless of the small number of studies, evidence is promising
Age	Strong confidence (high level of evidence)	One randomized controlled trials was included, one low-quality qualitative study and eleven observational studies initially rated low-quality and upgraded to high-quality using the GRADE system were included. All included studies showed a consistent positive effect on sustainable RTW
Gender	Very low confidence (very low level of evidence)	Despite some randomized control trials and large sample sizes, there were conflicting results regarding effects of gender on sustainable RTW for both men and women. Some studies suggest men RTW more sustainably than men, while a few studies suggest otherwise. It, therefore, suggest that it is possible that the effect of gender on sustainable RTW is influenced by an interaction of some factors for both sexes. However, it is unclear what specific factors are involved. Hence the need for further research in this area
Education	Moderate confidence (moderate level of evidence)	Five observational studies upgraded to high quality study based on the GRADE criteria. There were consistent positive effects across all five studies
Length of absence	Moderate confidence (moderate level of evidence)	Four studies with one randomized controlled trial and three observational studies upgraded to high quality study based on the GRADE criteria. There were consistent positive effects across all four studies
Job contract/security	Very Low confidence (very low level of evidence)	Only two observational studies upgraded to high quality based on the GRADE criteria. More studies would be necessary to draw strong conclusions on its effects on sustainable RTW
Support from leaders plays a role in facilitating sustainable RTW for employees with common mental disorders (CMDs)	Strong confidence (high level of evidence)	There were six randomized controlled trials, four and seven high quality mixed studies and qualitative studies according to the CERqual criteria respectively and 1 low quality qualitative studies. Thirteen out of sixteen low quality observational studies were upgraded to high quality studies based on the GRADE system, while three of the remaining observational studies maintained its low quality grade. Evidence presented is considered promising
Support from co-workers plays a role in facilitating sustainable RTW for employees with common mental disorders (CMDs)	Strong confidence (high level of evidence)	Five high-quality qualitative studies, three high-quality mixed study and one low-quality qualitative study based on the CERqual criteria were included. Six observational studies initially rated low quality using the GRADE system were included. Five of which were upgraded to high-quality studies using the GRADE upgrade criteria and one of which was graded low quality. Although there were no randomized control trials, twelve out of the fifteen included studies showed consistent positive effects on sustainable RTW
Job-crafting plays a role in facilitating sustainable RTW for employees with common mental disorders (CMDs)	Very low confidence (very low level of evidence)	Only two observational studies upgraded to high quality based on the GRADE criteria. More studies are required to build strong evidence base in this area
Personal characteristics play a role in facilitating sustainable RTW for employees with common mental disorders (CMDs)		
Attitude	Strong confidence (high level of evidence)	Only one randomized control trial, one high-quality qualitative studies and two high-quality mixed methods studies based on the CERqual criteria were included. Ten observational studies initially rated low quality using the GRADE system were included. Seven of which were upgraded to high-quality studies using the GRADE upgrade criteria and three of which were graded low quality. Twelve studies produced promising evidence with consistent positive effects on sustainable RTW
Self-efficacy	Moderate confidence (moderate level of evidence)	Only one randomized control trial and six observational studies upgraded to high-quality studies using the GRADE upgrade criteria and three of which were graded low quality. Apart from one observational study, all six studies produced promising evidence regarding the effects of self-efficacy on sustainable RTW
Age	Strong confidence (high level of evidence)	Ten observational studies upgraded to high-quality studies using the GRADE upgrade criteria. One of which was ranked low quality. Studies produced promising evidence of the effects of age on worker’s ability to RTW sustainably after ill-health
Gender	Very low confidence (very low level of evidence)	There were conflicting results regarding the effects of gender on sustainable RTW for both men and women. Some studies suggest men RTW more sustainably than men, while a few studies suggest otherwise. It, therefore, suggest that it is possible that the effect of gender on sustainable RTW is influenced by an interaction of some unknown factors for both sexes. Hence the need for further research in this area
Education	Low confidence (low level of evidence)	Four observational studies. Three of which were upgraded to high quality and one maintained the initial low quality rating based on the GRADE criteria. Although all three studies showed a consistent positive effects on sustainable RTW, evidence is not considered strong
Economic status/income	Very low confidence (very low level of evidence)	Only two observational studies. One of which was upgraded to high quality based on the GRADE criteria and the other graded low. More studies would be necessary to draw strong conclusions on its effects on sustainable RTW
Length of absence	Very low confidence (very low level of evidence)	Only two observational studies upgraded to high quality based on the GRADE criteria. More studies would be necessary to draw strong conclusions on its effects on sustainable RTW
Job contract/security	Very low confidence (very low level of evidence)	Only two observational studies upgraded to high quality based on the GRADE criteria. More studies would be necessary to draw strong conclusions on its effects on sustainable RTW
Sustainable RTW for employees with musculoskeletal disorders (MSDs) is dependent on the interplay between multiple personal and social factors	Moderate confidence	Only one low quality randomized controlled trial was included. Two mixed studies and one qualitative study graded high quality using the CERqual criteria were also included. Out of thirteen observational studies included, ten were upgraded to high quality studies as a result of meeting GRADE criteria. However, the remaining three maintained the low quality grade assigned to it by the criteria as a result of the study design. Results suggest that sustainable RTW for employees with MSDs is dependent on an interplay of personal and social factors
Sustainable RTW for employees with common mental disorders (CMDs) is dependent on the interplay between multiple personal and social factors	Moderate confidence	Two randomized controlled trials were included in this evaluation. Four mixed studies graded high quality using the CERqual criteria were also included. Out of twelve observational studies included, eight were upgraded to high quality studies as a result of meeting the GRADE criteria. However, the remaining four maintained the low quality grade assigned to it by the criteria as a result of the study design. Generally, moderate quality studies were included in this study. Results suggest that sustainable RTW for employees with CMDs is dependent on an interplay of personal and social factors

Evidence showing common factors was organized using the International Classification of Functioning, Disability and Health (ICF) framework which is useful for assessing, describing and organizing information on health status and disability across different cultures and settings [46]. This framework was chosen because it has previously been used to evaluate RTW factors across different health conditions [18]. The ICF is composed of four broad components: personal (e.g. age, sex), the body functions and structures (e.g. disease injury-related), activity limitation (e.g. history of sickness absence, inability to perform some activities of daily living), and environmental factors (e.g. all factors related to working conditions, work environment, work support and accommodation). However, only personal and environmental factors of the ICF framework was taken into account in this review as evaluated factors did not extend to other components, apart from personal and social factors which are classed under each component respectively.

The level of confidence in the overall body of evidence for each personal and social factor in this review was rated in four categories of evidence (strong, moderate, low and very low confidence) developed from the GRADE and CERqual approach [47]. Where there is confidence that a factor impacted on sustainable RTW outcomes, evidence was rated ‘strong confidence’ (high level of evidence). ‘Moderate confidence’ (moderate level of evidence) suggests that an impact may occur but requires further investigation. Level of evidence was rated ‘low confidence’ (low level of evidence) where further research is required and although an effect may occur, there is less confidence than for evidence of ‘moderate confidence’. Where there was insufficient evidence to draw conclusions, evidence was rated ‘very low confidence’ (very low level of evidence). Confidence in the evidence was decided by discussion and consensus by the review authors, by balancing the number of studies showing an effect in a consistent direction and the quality of those studies as indicated in the sections below.

However, in practice, evidence was rated strong where at least 10 studies showed positive effects and no more than three studies showed null effects or where 28 or more studies showed positive effects, no more than five showed null effects and only 1 showed negative effects. Evidence was rated a moderate/low where at least four/three studies showed a positive effect and there were no studies showing null or negative effects. Where there were only two studies showing an effect, even if the effect was consistent, we deemed this a low level of evidence. Evidence was also rated as very low where there were inconsistent or contradictory results, which was where there were no more than four studies showing an effect in one direction and at least one study showing an effect in the other direction.

## Results

### Literature Search

Our search strategy identified 40,276 citations related to the research topic on the thirteen databases, online trial registers, grey literature, and reference lists. After duplicate entries, non-peer reviewed published work and studies of foreign languages were eliminated from combined citations from all the databases, 4385 citations were potentially eligible for inclusion in the review.

### Selection of Studies

**Fig. 1:**
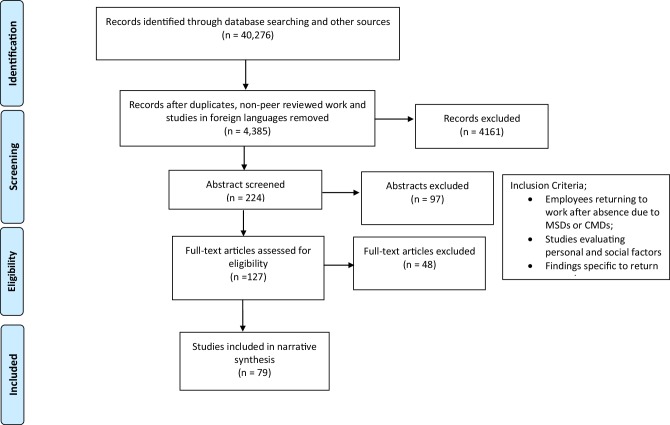
Flow chart of studies eligible for inclusion (Reproduced with permission from Moher et al. [71])

After removing 4161 citations at the title screening stage, 224 citations were left for the abstract screening. Of the 224 citations screened at this stage, 127 were left for the full-text screening stage. Out of 127 full-text articles retrieved, there was a unanimous agreement among the authors to include 58 papers and exclude 33 papers. However, there were disagreements between the authors on the eligibility of 36 studies evaluating the effectiveness of interventions on RTW outcomes. After further review of each of the 36 papers and in-depth discussions on its relevance or irrelevance, the authors finally agreed to include 21 citations (studies that took into account the impact of personal and/or social factors) and excluded 15 citations (studies with no personal and/or social factors in evaluation). Overall, of the 127 full-texted citations, a total of 48 papers were excluded based on not meeting the inclusion criteria. Seventy-nine articles were included in the final analysis. 55 studies of which reported RTW outcomes for workers sick-listed with MSD, while 45 studies reported RTW outcomes for workers sick-listed with CMDs. A flow chart (see Fig. 1) was developed to show the transparency of the selection process.

### Quality Appraisal

Out of the 18 randomized controlled trials that started out as very high-quality studies, one study was downgraded to low-quality as it did not take account of all confounding factors. Out of the 45 observational studies that started out as low-quality studies based on the standard GRADE rating, 42 were upgraded to high-quality studies as they met all the GRADE upgrade requirements. Based on the CERqual rating, out of the 16 qualitative and mixed studies included, one qualitative study was categorized as low-quality as a result of a lack of rigor in analysis and relationship between participants and researcher was not adequately considered [40]. The remaining 15 studies were categorized as high-quality because they fulfilled all the assessment criteria [40]. Taken as a whole, the quality of included articles reporting RTW outcomes for MSDs and CMDs did not affect the findings. Table 2 summarizes the main findings and the quality of the evidence supporting the main findings.

### Data Extraction

#### Study Characteristics

A total of 55 studies assessed the effects of personal and social factors on sustainable RTW due to MSDs. The study designs included randomized controlled trials (RCTs) (N = 12), observational studies (N = 33), qualitative studies (N = 9) and mixed methods studies (n = 1). Studies that examined whether there is evidence supporting suggestions that personal and social factors impact sustainable return to work (RTW) after ill-health due to CMDs totalled 45. The study designs included RCTs (N = 6), observational studies (N = 27), qualitative studies (N = 8) and mixed studies (N = 4). Workers in various occupational sectors returning to work after absence of at least two weeks due to MSDs and/or CMDs were represented in this review. Average age of study population ranged from 16 to 65 years. The majority of the studies (60 of 79) were conducted in Europe (Austria, Denmark, Finland, Ireland, Norway, Netherlands, Sweden, and United Kingdom). Five studies were undertaken in the United States, thirteen in Canada, and one each in Australia and China. Personal factors identified and evaluated included employee’s personal characteristics such as: attitude, self-efficacy, age, gender, education, economic status/income, length of sickness absence and job contract/security. Social factors identified and evaluated included support from leaders and co-workers and job-crafting practices.

### Evidence Synthesis

We reported findings from this review in two main categories; first, evidence on the effects of personal and social factors on sustainable RTW after ill-health due to MSDs or CMDs and second, evidence on personal and social factors common to both MSDs and CMDs. Personal and social factors that were common across MSDs and CMDs were determined based on the conclusions drawn from the evidence synthesis for both conditions. Outcomes were described in five groups (positive, negative, inconsistent, inconclusive and no effect). Common personal and social factors across MSDs and CMDs were deduced from consistent evidence from more than one study for both conditions. Where the majority of the outcomes (50% or more of the studies reporting a positive RTW outcome) in the review for each factor was in the same direction, evidence was considered consistent (see Table 3). Numerical representation of individual studies shown in the results is reported based on the evidence summary table presented in Online Resource 3.

**Table 3:** Common personal and social factors

Author	Condition	Sustainable RTW outcome
Positive outcomes		
*Personal factors*		
Positive attitude		
Anema 2003	MSDs	+
Bensen 2015	MSDs	+
Brouwer 2009	MSDs + CMDs	+
Brouwer 2010	MSDs	+
D’Amato 2010	MSDs + CMDs	+
Dionne 2013	MSDs	+
Dunstan 2013	MSDs	+
Ekbladh 2010	MSDs + CMDs	+
Ekbladh 2004	MSDs + CMDs	+
Heijbel 2006	MSDs + CMDs	+
Hoefsmit 2014	MSDs + CMDs	+
Labriola 2006	MSDs	+
Laisne 2013	MSDs	+
Opsahl 2016	MSDs	+
Reiso 2003	MSDs	+
Wahlin 2012	MSDs + CMDs	+
Ekberg 2015	CMDs	+
Martin 2015	CMDs	+
Nielsen 2013	CMDs	+
Van Oostrom 2009	CMDs	+
Volker 2015	CMDs	+
Self-efficacy		
Brouwer 2009	MSDs + CMDs	+
Brouwer 2010	MSDs + CMDs	+
D’Amato 2010	MSDs + CMDs	+
Huijs 2012	MSDs	+
Lagerveld 2010	CMDs	+
Van Beurden 2015	CMDs	+
Volker 2015	CMDs	+
Younger age		
Crook 1994	MSDs	+
D’Amato 2010	MSDs + CMDs	+
Gallagher 1989	MSDs	+
Heijbel 2006	MSDs + CMDs	+
Heijbel 2013	MSDs + CMDs	+
Huijs 2012	MSDs + CMDs	+
Laisne 2013	MSDs	+
Lederer 2012	MSDs	+
Lydell 2009	MSDs	+
Reiso 2003	MSDs	+
Steenstra 2009	MSDs	+
Stoltenberg 2010	MSDs + CMDs	+
Wahlin 2012	MSDs + CMDs	+
Engstrom 2007	MSDs	+
Lammerts 2016	CMDs	+
Roelen 2012	CMDs	+
Volker 2015	CMDs	+
Higher education		
D’Amato 2010	MSDs + CMDs	+
Huijs 2012	MSDs	+
Lydell 2009	MSDs	+
Muijzer 2011	MSDs + CMDs	+
Wahlin 2012	MSDs + CMDs	+
Ekberg 2015	CMDs	+
Inconsistent outcomes		
Gender		
De Rijk 2008	MSDs + CMDs	+/−
Lederer 2012	MSDs	+/−
Lydell 2009	MSDs	+/−
Opsahl 2016	MSDs	+/−
Crook 1994	MSDs	+/−
Johansson 2006	CMDs	+/−
Roelen 2012	CMDs	+/−
Volker 2015	CMDS	+/−
Laisne 2013	MSDs	+/−
No effects		
Positive attitude		
Brouwer 2010	CMDs	None
De Vries 2014	CMDs	None
Self-efficacy		
Huijs 2012	CMDs	None
Inconclusive outcomes		
Low economic status/income		
Lammerts 2016	CMDs	+/?
Roelen 2012	CMDs	+/?
Short-term length of absence		
Gallagher 1989	MSDs	+/?
Heijbel 2006	MSDs + CMDs	+/?
Lydell 2009	MSDs	+/?
Steenstra 2009	MSDs	+/?
Engstrom 2007	CMDs	+/?
Temporary and insecure job contract		
Huijs 2012	MSDs + CMDs	+/?
Lederer 2012	MSDs	+/?
Lammerts 2016	CMDs	+/?
Positive outcomes		
*Environmental factors: social factors*		
Support from leaders		
Ahltrom 2013	MSDs + CMDs	+
Anema 2003	MSDs	+
Baril 2003	MSDs	+
Bernacki 2000	MSDs + CMDs	+
Brouwer 2009	MSDs + CMDs	+
Brouwer 2010	MSDs	+
Brouwer 2011	MSDs	+
Bultmann 2009	MSDs	+
Burtler 2007	MSDs	+
D’Amato 2010	MSDs + CMDs	+
Dionne 2013	MSDs	+
Durand 2000	MSDs	+
Ekbladh 2004	MSDs + CMDs	+
Franche 2007	MSDs	+
Friesen 2001	MSDs + CMDs	+
Haugli 2011	MSDs + CMDs	+
Haveraaen 2016	MSDs	+
Heijbel 2013	MSDs + CMDs	+
Hoefsmit 2014	MSDs + CMDs	+
Hu 2014	MSDs	+
Janssen 2003	MSDs + CMDs	+
Jakobsen 2014	MSDs	+
Jensen 2012	MSDs	+
Labriola 2006	MSDs	+
Laisne 2013	MSDs	+
Loisel 1997	MSDs	+
Lysaght 2008	MSDs + CMDs	+
Muijzer 2011	MSDs + CMDs	+
Selander 2015	MSDs + CMDs	+
Shaw 2008	MSDs	+
Shiri 2011	MSDs	+
Steenstra 2006	MSDs	+
Tjulin 2011	MSDs + CMDs	+
Vermeulen 2011	MSDs	+
Wainwright 2013	MSDs	+
Andersen 2014	CMDs	+
Arends 2013	CMDs	+
Bond 2001	CMDs	+
De Vries 2014	CMDs	+
Hatchard 2012	CMDs	+
Karlson 2010	CMDs	+
Karlson 2014	CMDs	+
Martin 2015	CMDs	+
Nieuwenhuijsen 2004	CMDs	+
Post 2005	CMDs	+
Poulsen 2014	CMDs	+
Stahl 2014	CMDs	+
Tehiala 2013	CMDs	+
Van Beurden 2015	CMDs	+
Support from co-workers		
Brouwer 2009	MSDs + CMDs	+
Brouwer 2010	MSDs	+
Brouwer 2011	MSDs	+
D’Amato 2010	MSDs + CMDs	+
Dunstan 2013	MSDs	+
Ekbladh 2004	MSDs + CMDs	+
Friesen 2001	MSDs + CMDs	+
Haugli 2011	MSDs + CMDs	+
Haveraaen 2016	MSDs	+
Jakobsen 2014	MSDs	+
Labriola 2006	MSDs	+
Laisne 2013	MSDs	+
Lysaght 2008	MSDs + CMDs	+
Selander 2015	MSDs + CMDs	+
Tjulin 2011	MSDs + CMDs	+
De Vries 2014	CMDs	+
Hatchard 2012	CMDs	+
Nielsen 2013	CMDs	+
Stahl 2014	CMDs	+
Negative outcomes		
Support from leaders		
Post 2005	MSDs	−
Ekberg 2015	CMDs	−
No effects		
Support from leaders		
Arnetz 2003	MSDs	None
Besen 2015	MSDs	None
Verbeek 2002	MSDs	None
Wahlin 2012	MSDs	None
Nielsen 2013	CMDs	None
Brouwer 2010	CMDs	None
Van Oostrom 2009	CMDs	None
Van Oostrom 2010	CMDs	None
Volker 2015	CMDs	None
Support from co-workers		
Besen 2015	MSDs	None
Post 2005	MSDs + CMDs	None
Brouwer 2010	CMDs	None
Volker 2015	CMDs	None
Inconclusive outcomes		
Job crafting		
Bond 2001	CMDs	+/?
Johansson 2006	CMDs	+/?
Jakobsen 2014	MSDs	+/?
Krause 2001	MSDs	+/?
Marhold 2001	MSDs	+/?

Where sustainable RTW outcomes is represented as positive (+), negative (−), no effect (none), inconsistent (+/−) and inconclusive (+/?)

### Evidence on the Effects of Personal and Social Factors on Sustainable RTW After Ill-Health

Included studies presented a varied level of evidence ranging from strong to very low on the effects of personal and social factors on sustainable RTW for MSDs and CMDs.

#### Attitude

##### MSDs

Three very high-quality studies (18, 34, 58), nine high-quality studies (8, 10, 11, 16, 20, 32, 44, 61, 78) and four low-quality studies (3, 23, 24, 46) provided a strong level of evidence supporting the helpful effects of a positive attitude towards work and the RTW on sustainable RTW.

##### CMDs

While one very high-quality study (18) and one high quality study (11) did not find any association between attitude and sustainable RTW, three very high-quality studies (34, 53, 74), six high-quality studies (10, 16, 32, 55, 77, 78) and three low-quality studies (22, 23, 24) provided a strong of evidence that people with a positive attitude are more likely to RTW sustainably than those with a negative attitude towards work and the RTW process.

#### Self-Efficacy

##### MSDs

In four high-quality studies (10, 11, 16, 36), sustainable RTW was associated with self-efficacy, providing moderate level of evidence that employees with a high sense of self-efficacy are likely to RTW sustainably than those with a low self-efficacy.

##### CMDs

One very high-quality study (72) and seven high-quality studies (10, 11, 16, 36, 45, 77) examined the effects of self-efficacy. Apart from one study (36), all studies provided moderate evidence suggesting that employees with a high-self-efficacy during the RTW process have a greater likelihood of returning to work sustainably than those with a low sense of self-efficacy.

#### Age

##### MSDs

One very high-quality study (68), one low-quality study (46) and eleven high-quality studies (15, 16, 28, 32, 33, 36, 48, 50, 61, 69, 78) provided a consistent positive effect of age on ability to RTW sustainably, providing a strong level of evidence showing that younger employees of age ranged between 16 and 45 years have a higher probability of remaining at work after return than the older employees.

##### CMDs

Across all nine high-quality studies (16, 25, 32, 33, 36, 47, 69, 77, 78) and one low-quality study (62), there is a strong level of evidence that being of a younger age (16–45 years) increases the likelihood of returning to work faster and sustainably compared to being of an older age which contributes to delay in recovery and lasting RTW.

#### Gender

##### MSDs

Two high-quality studies (15, 48) reported sustainable RTW in women, while one very high-quality study (58) and three high-quality studies (17, 48, 50) reported sustainable RTW in men. Based on these inconsistencies in the findings, it is unclear which gender of the two is more likely to return to work sustainably after an absence spell, thus suggesting the need for further research. Hence, the evidence presented is considered very low.

##### CMDs

Two high-quality studies (40, 72) suggests the likelihood of women returning to work more sustainably than men, while two high-quality studies (17, 40) and one low-quality study (62) presented evidence of more sustainable RTW in men. Therefore, as with MSDs, there are inconsistencies in the evidence on sustainable RTW and gender, and the level of evidence is considered very low.

#### Education

##### MSDs

Five high-quality studies (16, 36, 50, 54, 78) provided a moderate level of evidence that workers with a higher level of education are more likely to RTW sustainably than those with lower levels of education.

##### CMDs

One low-quality study (22) indicated the positive impact of a low educational level on sustainable RTW. However, results from three high-quality studies (16, 54, 78) provided contrary evidence suggesting that employees with a higher educational level are more likely to engage with the RTW process which impacts positively on a sustainable RTW. There is therefore very low level of evidence of an association between high educational level and sustainable RTW.

#### Economic Status/Income

##### MSDs

There were no studies found to evaluate the effects of economic status/income on MSDs.

##### CMDs

Results from one high-quality study (47) and one low-quality study (62) indicated that RTW was not a result of recovery from ill-health. Instead, it was influenced by employee’s low income/economic status. However, the level of evidence provided is very low as a result of the limited number of studies reporting the effects of economic income/status on RTW outcomes.

#### Length of Absence

##### MSDs

One very high-quality study (68) and three high-quality studies (28, 32, 50) provided results indicating an effect of length of sickness absence, suggesting that to an extent, a short-term absence from work is likely to increase chances of a sustainable RTW. Therefore, there is a moderate level of evidence for this effect.

##### CMDs

Findings from two high-quality studies (25, 32) showed that the chances of sustainable RTW is heightened for employees out on a short-term sick-leave for not more than a year compared to those out of work on a long-term basis. Therefore, there is a very low level of evidence to support the impact of length of absence on sustainable RTW outcomes.

#### Job Contract/Security

##### MSDs

In two high-quality studies (36, 48), having a temporary and insecure job contract or working less than 40 h/week was associated with a sustainable RTW, providing a very low of evidence for this effect, with limited studies to draw definitive conclusions on lasting impacts of return.

##### CMDs

Two high-quality studies (36, 47) investigating the effects of an employee’s job contract/security on sustainable RTW showed that employees who are on a temporary or contract job and working less than 40 h/week are likely to RTW more sustainably regardless of ill-health condition compared to those with a permanent and secure working contract. This evidence was considered very low as a result of the few numbers of studies investigating this effect.

#### Support from Leaders

##### MSDs

Forty studies evaluated the role of support from leaders. Fifteen very high-quality studies (6, 13, 19, 27, 30, 34, 38, 49, 51, 63, 65, 67, 71, 76, 79), sixteen high-quality studies (1, 10, 11, 12, 14, 16, 21, 26, 31, 33, 35, 37, 39, 44, 54, 64) upgraded based on the GRADE criteria and 4 low-quality studies (3, 7, 24, 46) found sustainable RTW to be facilitated by support from leaders. Two very high-quality studies (5, 75) and two high-quality studies (8, 78) showed no effects of support from leaders on RTW outcomes. One high quality study (59) showed a negative effect of support from leaders on RTW outcomes. However, evidence synthesis provides a strong level of evidence suggesting that support from leaders does play a role in sustainable RTW outcomes in most instances.

##### CMDs

Fifteen very high-quality studies (2, 4, 18, 27, 29, 30, 34, 41, 51, 53, 60, 63, 66, 71, 72), eleven high-quality studies (1, 9, 10, 16, 33, 37, 42, 54, 57, 59, 70) and two low-quality studies showed that workers perceived support from leaders as a positive influence on their ability to RTW sustainably. Three very high-quality studies (56, 73, 74) and two high-quality studies (11, 77) indicated no effects on sustainable RTW. One low-quality study (22) indicated a negative effect on sustainable RTW due to support from leaders. There is therefore strong evidence suggesting the impact of support from leaders on sustainable RTW.

#### Support from Co-workers

##### MSDs

Six very high-quality studies (27, 30, 38, 51, 63, 71), seven high-quality studies (10, 11, 12, 16, 20, 31, 44) and two low-quality studies (24, 46) suggest that support from co-workers may have positive effects on sustainable RTW. However, one very high-quality study (59) and one high-quality study (8) provided evidence of no such association. Therefore, there is strong evidence that support from co-workers plays a role in sustainable RTW outcomes.

##### CMDs

Eight very high-quality studies (18, 27, 29, 30, 51, 56, 63, 71), two high-quality studies (10, 16) and two low-quality study (24, 66) provided results regarding the good effects of support from co-workers on sustainable RTW. However, findings from three high-quality studies (11, 59, 63) suggest that support from co-workers has no effects on sustainable RTW outcomes. Regardless, there is strong evidence suggesting that taking into account the effects of support from co-workers during the RTW process might be beneficial.

#### Job Crafting

##### MSDs

Two very high-quality studies (38, 52) and one high-quality study (43) provided evidence suggesting that sustainable RTW may be dependent on the employee’s ability to optimize their jobs by applying job crafting practices. However, evidence was considered low as studies were too few to draw a definite conclusion.

##### CMDs

Only two high-quality studies (9, 40) evaluating the effects of job crafting practices indicated positive effects on RTW outcome, however, providing a very low level of evidence with limited studies to conclude on its impact on a sustainable RTW.

### Evidence on Common Personal and Social Factors

A summary of the evidence on common personal and social factors associated with sustainable RTW outcomes is presented in Table 3.

#### Common Personal and Social Factors with Positive and Negative Sustainable RTW Outcomes

There was a consistently positive effect of four personal and two social factors on sustainable RTW outcomes for people sick-listed with MSDs and CMDs. Personal factors included a positive attitude, high self-efficacy, employees of a younger age and a high educational level. Social factors included support from leaders and co-workers.

Even though support from leaders showed a consistently positive effect on sustainable RTW among people sick-listed with MSDs and CMDs in most studies, two studies reported the opposite relationship for both MSDs and CMDs (59, 22). In these studies, contrary to evidence found in a large number of studies, low supervisory support facilitated a sustainable RTW. However, external factors outside of the workplace had an impact on these outcomes.

#### Common Personal and Social Factors with Inconsistent Sustainable RTW Outcomes

Gender was the only personal factor across all included studies that produced inconsistent effects on sustainable RTW for people with MSDs and CMDs. Reports for MSDs RTW outcomes in one study indicated the possibility of women returning more sustainably than men (15). One study showed a sustainable RTW for both genders (48). While three studies recorded sustainable RTW for men only (17, 50, 58). Reports for CMDs RTW outcomes also showed the same inconsistencies in findings. One study recorded more sustainable RTW among women (77) and two studies considered men more likely to RTW sustainably (17, 62). The contradiction in these results suggests the influence of another factor or factors on these RTW outcomes for both genders, hence the need for further research in this area.

#### Common Personal and Social Factors with No Effect and Inconclusive Sustainable RTW Outcomes

Personal factors showing inconclusive sustainable RTW for people with MSDs and CMDs included short-term sickness absence and temporary and insecure job contract. Across both MSDs and CMDs, the effect of job crafting was inconclusive because included studies were too few to infer firmly on their impact, thus warranting the need to investigate further on these effects.

We found a few studies where positive attitude (11, 18), a high self-efficacy (36), support from leaders (5, 8, 75, 78, 56, 11, 73, 74, 77) and support from co-workers (8, 59, 11, 77) showed no effects on RTW outcomes. However, further investigation of these null outcomes showed the influence or absence of other factors which may have impeded expected RTW outcomes. For example, in three studies presence of a positive attitude towards work and the RTW process (25, 43) and a high self-efficacy (44) failed to impact on RTW outcomes due to the notable absence of social support in the workplace which was in other studies associated with expected outcomes.

## Discussion

The main aim of this review was to assess the impact of personal and social factors on sustainable RTW after ill-health due to MSDs and CMDs and to identify commonalities of effects of these personal and social factors between both conditions. Across the literature on facilitators and barriers of RTW, personal and social factors may include a range of concepts not evaluated in this review. However, the evidence presented in this review is only limited to the factors identified in the included studies to influence sustainable RTW outcomes. Overall, sustainable RTW was evident across all RTW interventions or measures involving the personal and social factors evaluated. Effects of assessed personal and social factors were shared across both MSDs and CMDs, and the results were generally in the same direction. This review highlights that personal and social factors play vital roles in facilitating or impeding sustainable RTW after ill-health due to MSDs and CMDs, aligning with Alavi and Oxley’s [6] findings. This may suggest that taking into account employees’ personal and social factors when implementing RTW interventions or programmes will be more beneficial on RTW than modifying or adjusting their job role alone on RTW.

Findings from this review indicate that the effects of personal and social factors are likely to be correlated. Evidence suggests that sustainable RTW may be facilitated by employees having a positive attitude towards work and the RTW process and a high self-efficacy which are boosted by support from leaders and co-workers during the RTW process. This inference is from results from a few studies where the effects of attitude [24, 48] and self-efficacy [49] on sustainable RTW for people with CMDs was inhibited as a result of an absence of support at the workplace. According to Haveraaen et al. [50], high support from leaders and co-workers could improve the self-confidence and optimism of the returning worker, thus making them feel valued and worthy. This suggests that it is social support that may lead to better attitude and self-efficacy and therefore to better RTW outcomes. However, it is also possible that leaders and co-workers are more inclined to support employees who have a positive attitude towards work and the RTW process and a high confidence in their job competence which in turn impacts on sustainable RTW. The nature of the interaction between these factors is still unclear and should be studied in more detail in the future. Although support in the workplace showed a positive influence on sustainable RTW, however, across two studies that evaluated support from leaders [51, 52] among individuals with CMDs and MSDs respectively, the evidence did not align with these other findings. Instead, sustainable RTW was facilitated irrespective of the low level of support during the RTW process. These unusual findings can be explained that in these instances, workers returned to work despite being ill in order not to lose their jobs [51, 52].

Job crafting could be beneficial to employees with MSDs and CMDs returning to work after a period of absence. Findings suggested that its effect on sustainable RTW was associated with supportive interactions at the workplace [53–56]. Employees who felt supported by their line managers and co-workers and were given the opportunity to plan their jobs during the RTW process were more likely to have a high sense of control over their jobs. As a result, they were able to redesign their job tasks in a way that satisfied them, which in turn impacted sustainable RTW outcomes. These conclusions support Wang et al.’s [57] and McClelland et al.’s [58] notion of support as an essential antecedent to the effectiveness of job-crafting. They assert that where leaders and co-workers work with employees in a supportive capacity, it is likely to increase the employee’s motivation and thereby stimulate their job crafting abilities. However, evidence for the effects of job crafting on sustainable RTW is inconclusive as only a few numbers of studies have investigated this association, as such, it is unclear if other unknown factors have influenced these observed outcomes. Future research should, therefore, investigate the relationship between support from leaders and co-workers and employee’s ability to craft their jobs and how that impacts sickness absence. Though included studies did not investigate the impact of collaborative job crafting (team-level job crafting), it might also be beneficial to probe further the effects of collaborative job crafting on RTW.

The effects of younger age, higher education, low economic status, a short-term length of absence, and a temporary and insecure job contract produced evidence suggesting its positive impact on sustainable RTW. Cancelliere et al.’s [18] findings also identified higher education levels and socioeconomic status as prognostic factors associated with positive RTW outcomes among people with MSDs and CMDs. This review thus verifies that association, suggesting the need to take into account employee’s varied personal characteristics when implementing RTW measure for a more sustainable outcome.

Across the studies, younger aged workers were more likely to RTW sustainably than older employees, corresponding with Cornelius et al.’s [59] findings. Employees of the older workforce are considered more susceptible to ill-health, as such if they RTW, they had a higher probability of becoming ill again. Sustainable RTW outcomes were more prevalent among employees of a high educational level than employees of a lower educational level in all studies. The reviewed studies discovered that participants who were more willing to participate in RTW interventions were highly educated in all cases, had high quality jobs, stronger job resources, and higher expectations. According to Piha et al. [60], people with higher education levels are accorded more understanding and knowledge about health-related factors including health behaviours which helps them make healthier decisions in their everyday life and lifestyle which impacts positively on RTW outcomes. The likelihood of sustainable RTW was further increased among people with low income/economic status, temporary/contract jobs. Employees in these categories showed that it was more important to maintain their source of income and keep their job, hence the decision to RTW faster regardless of their health condition to avoid loss of employment as a result of extended absence. Positive effects on sustainable RTW were also identified among employees on a short-term absence from work [17, 61].

These conditions raise concerns about the risk of decisions to RTW while not fully recovered may pose to employees and the cost it may incur to employers. According to Whysall et al. [62], if RTW is not managed appropriately, this risk is likely to exacerbate existing medical conditions, impair quality of life, invite feelings of ineffectiveness at work and produce a cumulative psychological burden with consequences. As some personal factors like age or gender are not adjustable, employers have the responsibility to ensure they understand employees’ conditions and provide adequate preventive measures to support them on RTW.

Results on the effects of gender were inconsistent. Previous studies have often identified men as the most likely to RTW sustainably [63–65]. Men are considered to be more willing to engage in the RTW process because they attribute more importance to their work [66]. However, in this review, we found some studies that reported that women were more likely to RTW more sustainably than men [67, 68], while other studies showed that men were more likely to RTW sustainably [63–65, 69]. The discrepancies in these findings suggest the influence of additional factors on RTW outcomes. It is, therefore, unclear if the effects of gender vary based on factors such as the sector these individuals work in or the organizational culture in the workplace. Moreover, it is possible that factors that influence RTW outcomes for men and women vary, hence the need to conduct further research on these effects to understand precisely the factors that affect RTW outcomes for both men and women.

This review revealed common personal and social factors associated with a positive, sustainable RTW outcome for people sick-listed with both MSDs and CMDs. They included a positive attitude, high self-efficacy, younger age, higher education, and support from leaders and co-workers. Rather than tackling MSDs and CMDs separately, recognizing these common factors will be a beneficial step for employers in implementing a holistic RTW approach/intervention for both conditions. According to Naylor et al. [27], if the integration of mental and physical health does not form a significant component of programmes, it would be a significant missed opportunity.

### Strengths, Limitation and Gaps in Evidence

The review process had the aim of being thorough, transparent and reproducible, and the critical appraisal method allowed for the inclusion of high-quality papers. A wide range of study designs was included with the intention of avoiding an overlook of evidence that is often considered too weak for inclusion. However, it is possible that the selection approach adopted in this process could have increased the risk of selection bias which may have resulted in the exclusion of potentially relevant studies. It is also possible that some studies that would have been relevant to this review have not been identified because of them being unpublished.

One of the strengths of this review lies in the methodological build-up. Reporting the effects of a variety of personal and social factors and identifying the commonalities between conditions may have introduced a degree of complexity to the analytical process. Harvest plots were developed for ease of synthesis and visual display of evidence to support competing hypotheses about the impact of evaluated factors on sustainable return to work for both conditions separately. This graphical method of synthesizing findings adapted from Thomas et al. [44] seemed very useful to synthesize evidence across multiple sources.

The review revealed several gaps in the currently available evidence. Most notable is a lack of sufficient literature evaluating the effects of job crafting, economic status, length of absence and job contract/security on sustainable return to work, making it challenging to draw confident conclusions. Hence, it would be useful to conduct further research in these areas to aid clear conclusions regarding its effects. Additionally, this review identified inconsistent results surrounding the impact of gender on sustainable RTW, suggesting the influence of other factors.

## Conclusion

Alavi and Oxley [6] assert that when research concentrates more on learning about factors associated with sustainable RTW, significant gains in RTW programs will be achieved. This review addresses this call by contributing evidence towards understanding the role of various factors that facilitate a sustainable RTW for workers sick-listed with MSDs and CMDs.

Personal and social factors play a role in facilitating sustainable RTW after ill-health due to MSDs and CMDs. However, sustainable RTW does not appear to be the result of a single factor. Instead, sustainable RTW seems to be influenced by an interplay of multiple factors. Here the most consistent evidence for sustainable RTW was found for support from leaders and co-workers, positive attitude, high self-efficacy, younger age and higher education levels.

The results of this review indicate that existing RTW programmes need to encourage supportive interactions between leaders and co-workers and returning workers during the RTW process [70], especially as this could have a direct effect on sustainable RTW, as well as an indirect effect through enhanced returners’ attitudes toward work and self-efficacy. Also, it suggests a role for the state in encouraging employers to implement RTW strategies that factor in management (and other) support and to work on developing positive attitude and self-efficacy among returning workers. Although RTW takes place within a complex system involving employing organizations and the healthcare system, given the consistent evidence of the role line managers play in sustainable RTW, we recommend that policymakers consider ways to provide guidance for employers. Guidance could: outline the supportive role of line managers and other key workplace professionals (e.g., human resources professionals, occupational health providers) during the return to work process; train these key workplace professionals on the return to work process and how to effectively manage and support returning workers; and outline ways to facilitate line managers in providing necessary support. Promoting a culture of support at the workplace is essential, a culture that makes returning workers feel valued, worthy and not necessarily blamed for absence, as the former would improve work attitudes and ease the transition back to work.

## Electronic supplementary material

Below is the link to the electronic supplementary material.

Supplementary material 1 (DOCX 767 KB)
