# Supplementary material for: Sustainable Return to Work: A Systematic Review Focusing on Personal and Social Factors
Source: J Occup Rehabil. 2019 Feb 15;29(4):679–700. doi: 10.1007/s10926-019-09832-7 (PMC6838034; doi:10.1007/s10926-019-09832-7)
Supplement: Supplementary file 1 — Supplementary material 1 (DOCX 767 KB) [file 10926_2019_9832_MOESM1_ESM.docx]

# SUSTAINABLE RETURN TO WORK AFTER ILL-HEALTH: A SYSTEMATIC REVIEW FOCUSING ON PERSONAL AND SOCIAL FACTORS.

***Journal of Occupational Rehabilitation***

Abasiama Bassey Etuknwa (MSc.) ^1^

Prof. Kevin Daniels (PhD.) ^2^

Dr. Constanze Eib (PhD.) ^3^

University of East Anglia, Norwich Business School. Norwich Research Park. Norwich. NR47TJ. United Kingdom.^1, 2^

Uppsala Universitet, Department of Psychology. Von Kraemers allé 1A och 1C 752 37 Uppsala, Box 1225 751 42 UPPSLA.^3^

[A.Etuknwa@uea.ac.uk](mailto:A.Etuknwa@uea.ac.uk) ^1^

# Online Resource 1: List of Electronic Databases Searched

| Name of Database | No. of Relevant Ref. Found | No. of Ref. Exported | Date Accessed | |
| --- | --- | --- | --- | --- |
| Business Source Complete | 1,188 | 11 | 05/01/2017 |  |
| CINAHL | 1,549 | 29 | 08/01/2017 |  |
| Cochrane Library | 352 | 2 | 01/11/2016 |  |
| EBOSCO Host | 1,138 | 8 | 08/01/2017 |  |
| JSTOR | 6,026 | 33 | 13/01/2017 |  |
| Medline (OVID) | 138 | 4 | 16/01/2017 |  |
| PsychINFO | 7,440 | 28 | 08/01/2017 |  |
| PubMed | 1,313 | 17 | 16/01/2017 |  |
| Scopus | 1,659 | 11 | 23/02/2017 |  |
| ScienceDirect | 12,025 | 42 | 10/03/2017 |  |
| SPORTDiscus | 6,999 | 12 | 10/03/2017 |  |
| Web of Science | 350 | 18 | 11/01/2017 |  |
| Wiley Online Library | 99 | 9 | 13/01/2017 |  |
| Total | 40,276 | 224 |  |  |

# Online Resource 2: Data Extraction Sheet

| Paper | Authors, Date, |
| --- | --- |
| Study design | Briefly state the type of study. |
| Pre/Post Follow-ups (Months) | How long study took and the number of follow-up post intervention. If appropriate, how participants were assigned to intervention groups. How many observations are there? |
| Dependent Variables | This describes the nature of sustainable return to work. Any mediator variables that transmit the effects of the intervention. |
| Independent Variables | This describes the nature of interventions or, for observational studies, the variables investigated |
| Population | Where is the data sourced from (does it overlap with other studies), age/gender/ethnic/disability etc.? |
| Sample Size | This simply states the total number of participants. Providing the sample size per group in intervention studies. |
| Data Collection Tool | This simply states the data collection tools (e.g. questionnaire, interview, etc.). |
| Response Rate | Simply state the response rate of participants to the intervention/study. Country/region of study. Age/gender/ethnic/disability etc.  composition if reported |
| Industrial Sector | State the industrial sector participating in the study. |
| Country | Country/region of study |
| Personal and Social factor included | This simply lists the personal and/or social factors evaluated in the studies. |
| Description of findings | What were the results with respect to sustainable return to work (including effect sizes, confidence intervals and their significance, for all relevant outcome) Were they positive or negative, or inconclusive, was causality established or demonstrated or just discussed/ suggested? |

# Online Resource 3: Evidence Summary Table

| Author/ Year | Study Design | Pre/Post Follow-Ups (Months) | Ill Health Condition | Dependent Variables | Independent Variables | Population | Sample Size | Data Collection  Tool | Response Rate | Industrial Sector | Country | Personal/ Social Factor Involved | Description of Findings |
| --- | --- | --- | --- | --- | --- | --- | --- | --- | --- | --- | --- | --- | --- |
| Ahltrom *et al*. 2013 | Prospective | 6 & 12 Months | MSDs, CMDs | Work ability and RTW | workplace rehabilitation, supportive conditions at work and time | Women aged 35-65 years on long-term sick-leave | N= 324 | Questionnaire | 72%, 60% | Human services organization | Sweden | Support from leaders | The results showed that individuals provided with workplace rehabilitation and supportive condition (e.g. influence at work, possibilities for development, degree of freedom at work, and meaning of work, quality of leadership, social support, and sense of community and work satisfaction) had significantly increased work ability and improved the RTW process for women on long-term sick leave. |
| Andersen *et al.* 2014 | Qualitative (Longitudinal) | 3 Interviews within just after randomization, 3 months after and 6-7 months after. | CMDs | RTW | Workability assessments, RTW activities. | Persons on sick leave for approximately 8 weeks due to stress or depression, who spoke and understood Danish. Average age of 44 years (range 23-61 years) | N= 18 | Interview | 94.4% at both 2^nd^ and 3^rd^ interview. | Various | Denmark | Support from leaders | At the last interview session, 11 participants had returned to work full time or part time or were no longer on sick leave. The workability assessment consultations and RTW activities could result in both motivation and frustration depending on the extent to which RTW professionals practiced an individual approach to sick listed persons. The individual approach seemed necessary for the realization of the positive potential in the RTW intervention. |
| Anema *et al*. 2003 | Randomized controlled trial | Within 3 months and after 3 months implementation. | MSDs | RTW | work design and organization, workplace and equipment design | Workers sick-listed between 2- 6 weeks due to LBP (male= 57.6%, mean age= 40.9) | N= 35 | Questionnaire | 78% | Health care & Social Security | Netherlands | Personal Characteristics (Attitude-Compliance), Support from leaders | Results suggests that participatory RTW programs was satisfactory and effective in stimulating a 66.7% RTW. It also suggests that compliance, satisfaction and acceptance of program by employees facilitates RTW. |
| Arends *et al.* 2014 | Cluster randomized controlled trial | 6 & 12 months | CMDs( stress, depression, anxiety, somatisation) | Recurrent SA | Mental health complaints | Workers between 18-63 years with an episode of SA due to CMD of at least 2 weeks. | N= 158 (N=80 in intervention group and N= 78 in control group) | Questionnaire | 94.4% & 64% (at 3 months for both intervention and control group respectively) | Health care | Netherlands | Support from leaders | Results suggest that support from leaders in the return to work process are effective in reducing the incidence of recurrent sickness absence. |
| Arnetz *et al.* 2003 | Prospective controlled trial | 0, 6 & 12 months | MSDs | RTW | Medical diagnose, days to rehab investigation, days to rehab plan, days to rehab cost, rehab cost, Number of sick days, age, gender and work hours | Employees of both gender diagnosed with a first or recurrent MSD. Mean age of 42.7 and 42.7 and Male/Female =31/41 and 26/39 in both intervention and reference group respectively. | N= 137 (N=65 in intervention group and N=72 in control group) | Standardized Nordic Questionnaire, Interview | 84.6% & 27.8% (for both the intervention and reference group respectively) | National Insurance | Sweden | Support from leaders | The odds ratio for returning to work in the intervention group was 2.5% (95% confidence interval 1.2-5.1) compared to the reference group. It is suggested that management of MSDs should to a greater degree focus on early RTW and building on functional capacity and employee ability. Allowing the case managers a more active role as well as involving ergonomist in workplace adaptation meetings might also be beneficial. |
| Baril *et al.* 2003 | Qualitative | N/A | MSDs | RTW | Personal and socio-demographic factors, beliefs, attitude and motivation. | All actors involved in the RTW process for workers with MSDs. (Injured worker, other actors in the workplace and those external to the workplace) | N= 55 (Manitoba)  N= 17 (Ontario)  N= 36 (Quebec) | In-depth semi-structured interviews by focus groups and document review. | N/A | Various | Canada | Support from leaders and Co-workers | Results from the study from injured workers suggests that characteristic influencing RTW success included personal and socio-demographic factors, beliefs and attitude and motivation. Human resources managers and health care professionals attributed worker’s motivation to their individual characteristic, while injured workers, worker representatives and health and safety managers described workplace culture and the degree to which workers’ well-being was considered as having a strong influence on workers’ motivation. RTW success was therefore attributed to labour management relations and top management commitment to Health and Safety. |
| Bernacki *et al.* 2000 | Longitudinal | 10 years | MSDs, CMDs | Early RTW | The number of non-lost time and lost time cases, time lost from work, and the number of restricted workdays, job analyses. | Employees with work-related conditions. | 1989 N= 16,212  1990 N= 16,851  1991 N= 17,022  1992 N= 17,136  1993 N= 17,771  1994 N= 18,282  1995 N= 19,565  1996 N= 20,921  1997 N= 21,016  1998 N= 22,156  1999 N= 28,518 | OSHA 200 Log database, Occupational injury clinic database, Health, safety and environmental department’s database. | - | Health Care | United States | Support from leaders | A significant decrease (55%) was observed in the rate of lost workday cases before versus after the return to work program. Furthermore, the number of lost workdays reduced from an average of 26.3 per 100 employees to 12.0 per 100 employee. The RTW initiative and the number of restricted duty days went from an average of 0.63 per 100 employees to 13.4 per 100 employees. The study suggests that a well-structured early RTW program is an integral part of a comprehensive effort to control the duration of disability associated with occupational injuries and illness. It also indicates that to be most effective, an early RTW program must include participation by medical providers, safety professionals, injured employees, and supervisors. It also suggests the effectiveness of RTW programs if it includes an individual trained in ergonomics to facilitate job placement process. |
| Besen *et al.* 2015 | Longitudinal | 3 time points (during initial visit to clinic, 7 days later and 3 months following initial visit). | MSDs (LBP) | RTW | Pain, catastrophizing, fear-avoidance beliefs, organizational support, RTW confidence, RTW expectations. | Participants with lumbar back pain with onset of less than 14 days. 18 -63 years. Average age= 38, Male= 54%, white= 72%, non-Hispanic = 78% | N= 241 | Questionnaire, Telephone interview, web-based survey, paper survey. | N/A | Health care | United States | Personal Characteristics (Attitude), Support from leaders and C-workers. | Results suggest that successful return to work after an episode of LBP directly related to RTW confidence and RTW expectations, while; Pain, catastrophizing, fear-avoidance beliefs, organizational support, and RTW confidence were indirectly related to the RTW outcomes. |
| Bond and Bunce. 2001 | Longitudinal Quasi-Experiment | 2 observational times. 1-year follow up | CMDs | Stress and SA reduction | Mental ill-health, SA, job control and self-rated performance, physical ill-health symptoms, job satisfaction. | Administrative employees in a UK central government department. Men= 61, Women= 36. 57% between 37 and 55 years, 6.2% over 55 years. 43% University graduates, 51% were middle management, 92% worked full-time and 67% were married or cohabitating. | N= 97 | Questionnaire | 56% in the PAR group and 53% in the control group. | Public | United Kingdom | Job Crafting, Support from Leaders | Study found that work re-organization (PAR) interventions stirred by leaders, increased job control which mediated improved participant’s mental health, significant reduction in sickness absence rates and increased self-rated performance. |
| Brouwer *et al.* 2009 | Prospective cohort | 10 months follow-up | MSDs, CMDs | Time to RTW | Attitude, severity of complaints, subjective norm (social support, social pressure) and self-efficacy (willingness to expend effort in completing a behaviour, persistence in the face of adversity and willingness to initiate behaviour) | Employees on sick leave with different types of symptoms. Absent for a maximum of 12 weeks. Men= 466, Women= 460. Aged 18-63 years. Mean age= 45.8 years. 33% of low level of education, 30% of medium level of education and 30% of high level of education. 352 reported MSDs, 235 reported mental symptoms and 256 reported other physical symptoms. | N= 926 | Questionnaire | 86% at baseline | Various | Netherlands | Personal characteristics (attitude, self-efficacy), Support from leaders and co-workers | Results suggest that median time to RTW was 160 days. In the Univariate analysis, all prognostic factors were significantly associated with time to RTW; work attitude, social support and the three subscales of self-efficacy. The final multivariate model with time to RTW as the predicted outcome included work attitude, social support and willingness to expend effort in completing a behaviour as significant predictive factors. |
| Brouwer *et al.* 2010 | Explorative (data from prospective 1-year cohort study) | 6-12 weeks after onset of sick-leave (baseline) and 10 months after listing sick. | MSDs, CMDs | Time to RTW across different health conditions | Perceived work attitude, self-efficacy and perceived social support | Workers on long-term sickness absence due to different types of symptoms. Absent for a maximum of 12 weeks. Workers with mental conditions such as Stress and depression or burnout, Workers with musculoskeletal conditions from back, upper and lower limb problems and workers with other physical conditions such as diseases of the circulatory, digestive, neurological and respiratory systems. reported other physical symptoms. | N= 862 (352= MSDs, 265= other physical conditions and 245= mental health conditions) | Questionnaire | 86% at baseline. | Various | Netherlands | Personal characteristics (Attitude, Self-Efficacy), Support from leaders and co-workers. | For workers out on musculoskeletal conditions, results showed that a good perceived work attitude, perceived support from supervisors, co-workers and other groups, and self-efficacy (willingness to expend effort in completing a behaviour) were significantly associated with time to RTW. While for workers out on mental health conditions, only self-efficacy (willingness to expend effort to complete a behaviour) was significantly associated with time to RTW. |
| Brouwer *et al.* 2011 | Prospective Cohort | 1,6,12 & 24 months post injury | MSDs (back or upper extremity) | RTW, RTW Self-efficacy. | RTW Self-efficacy, readiness for RTW, RTW status, SA duration and compensation characteristic, social support at work and health outcomes. | Workers who had filed a lost–time claim for back or upper extremity work–related MSDs. Only claims registered within 7 days post–injury were included | N= 632 at one month and N= 446 at 6 months. | Structured interviews, administrative database | 61% at 1 month. 71% at 6 months | Various | Canada | Support from leaders and co-workers | The factor analyses supported three underlying factors; obtaining help from supervisors, coping with pain and obtaining help from co-workers. The total variance for the three scales were 68% at 1 month follow up and 76% at 6 months follow up. With regards to construct validity, relationships of RTW self-efficacy with depressive symptoms, fear-avoidance, pain and general health were generally in the hypothesized direction However the hypothesis that less advanced stages of change on the readiness for RTW scale could not be completely confirmed. Only pain RTW self-efficacy was significantly associated with RTW status and duration of work disability. The strength of association between RTW self-efficacy and other constructs was stronger at 6 months post injury compared to 1-month post injury. |
| Bűltmann *et al.* 2009 | Randomized Controlled Trial | 12 months | MSDs | Cumulative SA hours | Work status, pain intensity, and functional disability | Participants absent from work for 4-12 weeks, who have a reimbursement request indicating LBP or MSD as the main cause of sick leave and are 18 -65 years of age. | N= 119  Intervention group= 68,  Control group= 51 | Questionnaire, Administrative data. | 97% in the intervention group and 92% in the control group. | Public | Denmark | Support from leaders | For the time interval 0-6 months, 6-12 months and the entire follow-up period, the number of SA hours was significantly lower in the intervention group compared to the control group. In conclusion, workers on sick leave for 4-12 weeks due to MSD who underwent the coordinated and tailored work rehabilitation intervention by an interdisciplinary team had fewer sickness absence hours than the controls. |
| Burtler *et al.* 2007 | Prospective Cohort | 1, 6 & 12 months | MSDs (LBP) | RTW | worker’s satisfaction and health care | Workers aged 18 and older who filed worker’s compensation claims for occupational back pain between January 1, 1999 and June 30, 2002. | N= 959 at one month follow up. N= 585 at six months follow up and N= 332 at 12 months follow up. | Survey, Interview | 51% at baseline, 87% at 1 month, 62% at 6 months and 42% at 1 year. | Education | United States | Support from leaders | Results suggests that worker’s satisfaction in the positive responses of their employers to their work-related injury claims is the most important influence on their stability in employment subsequent to onset of injury. Results show that although satisfaction with healthcare is influential, it is a much less important influence on patterns of employment than is a worker’s perception of the actions of his employer. |
| Crook and Moldofsky, 1994 | Prospective longitudinal | 3, 9, 15 & 21 months | MSDs | RTW or Remain on work at any point in time. | Gender, age, pattern of disability and RTW, LBP vs all other MSD pains | Workers who had sustained musculoskeletal injury at work and had not returned to work by 3 months post-injury. Male and female below age 60 (17-60). Mean age= 40.6, Males= 52.7 %, females= 47.3% | N=148 at 3 months, N= 120 at 9 months, N= 115 at 15 months and N= 108 at 21 months | Interview | 81% at 9 months, 95.8% at 15 months and 93.9% at 21 months. | Various | Canada | Personal characteristics | Results revealed that men are more likely to return to work earlier than women are. However, sustainability of RTW was more likely in women than in men. Workers aged 19-30 years had a higher probability of returning to work earlier, those aged 31-40 had a higher probability of remaining at work compared to workers aged 41-50 years. Results suggests that the probability of returning to work is dependent on the number of times work disability had recurred. Workers with shorter reoccurrence are more likely to remain at work. While workers with low back pain have a higher chance of reoccurrence compared to other musculoskeletal pains. |
| D’Amato and Zijlstra, 2010 | Longitudinal | 2 time waves (T1 & T2). Baseline and 6 months later. | MSDs, CMDs | RTW | Psychological factors (perceived health, well-being, self-efficacy, emotional exhaustion, depression, life events), Psychological aspects of the job (job stress, stress, work ability, work centrality), organizational policies for work resumption, experiences during the period of SA, RTW. | Worker in full-time employment previous to the period of absence, having been absent for no longer than 6 months. Male= 48.7%, Women= 51.3%. ≤35 and ≥55. | N= 1460 | Questionnaire | 73% at T2 | Various | Austria, Ireland, Finland, Netherlands and United Kingdom | Personal characteristics, Support from leaders and co-workers | Health improvement is necessary but it alone is not sufficient as precondition for RTW. Psychological factors (self-efficacy, depression) and organizational factors had the highest impact on RTW. Results suggest that age and level of education play a marginal role in predicting return to work. People’s beliefs and awareness were primary determinants of RTW. Arrangements made by the organization after a worker becomes absent to help RTW had a positive influence on RTW. |
| De Rijk *et al.* 2008 | Prospective Cohort | 7 time frames (T1, T2, T3, T4, T5, T6, & T7) over 13 months. | MSDs, CMDs | Initial and lasting RTW | Gender, RTW, lasting RTW, survival time to lasting RTW, self-rated health, reasons for reporting sick, presence of at least one long-term disease, early improvement and change in diagnosis. | Employees who reported sick for more than 1 month, who had visited their OP between 1^st^ May and 11^th^ November, 2000. Between 16-61 years, worked 20h per week or more. Male= 65, Women= 54 | N= 119 | Questionnaire, structured face to face interview, telephone interview | 56.6% at T1. 94.4% at the 1^st^ interview. | Various | Netherlands | Personal characteristics | Results suggest that men are more likely to have lasting RTW than women. Men with MSDs and no long-term disease were 3.5 times more likely to have lasting return to work then men with mental illness and at least one long-term disease. While women with and early improvement in health and no changes in diagnosis were 5.5 times more likely to have lasting RTW than women who did not experience improvement and whose diagnosis had changed. |
| De Vries *et al.* 2014 | Mixed | 2 phases. | CMDs (depression) | RTW | Employees, supervisors and occupational physicians. | Diagnosed with a major depressive disorder, have a paid job; have been on 100% sick leave for at least 1 year. | N= 60  (statement generation phase= 32, prioritization and categorization phase= 38) | Interview | 94% in phase 1 and 72% in phase 2 | Various | Netherlands | Personal characteristics (Attitude), Support from leaders and co-workers | Results suggest that **Person** (personality/coping problems, symptoms of depression and comorbid health problems, employees feeling misunderstood, and resuming work to soon), **Work** (troublesome work situation, too little support at work and too little guidance at work) **and Healthcare** (insufficient mental healthcare and insufficient care from occupational physician) were perceived as the main impeding factors for RTW after long-term absence related to major depressive disorder. |
| Dionne *et al.* 2013 | Qualitative | - | MSDs (back pain) | RTW | Obstacles and facilitators to RTW. | Workers suffering from back pain severe enough to limit work activities. Aged 18 - 60 years. Men= 14, Women= 5 | N= 19 | Focus group discussion, written list | 66.7% in Focus group 1 and 60% in Focus group 2 | Various | Canada | Support from leaders, Personal Characteristics | Results suggest that personal factors (knowledge of one’s limit and listening to one’s body and physical training), Understanding from employers during the RTW process and the possibility of gradual return to work were the main facilitators to RTW. |
| Dunstan *et al.* 2013 | Longitudinal | 3 stages. Baseline, one week after & 3 moths follow up. | MSDs | Factors influencing future work expectations. | Direct measure scales (Behavioural intervention, attitude, subjective norm, perceived behavioural control) and Indirect measure scales (behavioural beliefs, normative beliefs and control beliefs) | Workers with compensable work injury. Mean age= 43.7 years (18- 66.1 years), Male= 84 (53.2%) | N= 158 | Questionnaire, telephone interview | 35% | Healthcare | Australia | Personal characteristics (attitude), Support from co-workers | Results show that attitude, subjective norm and perceived behavioural control explained 76% of the variance in behavioural intention. While the expectation to RTW (Behavioural intention) explained, 51% of the variance in work participation at follow up. The strength of key influences on RTW varied according to employment status, but strong influences included availability of modified duties, social aspects of work, the opinion of the treating doctor, co-worker support, pain and functional limitations. |
| Durand *et al.* 2000 | Observational | 2 years | MSDs (back pain) | Stable RTW | Quality of life, specific back disability, pain intensity, fear and avoidance beliefs, absenteeism and depression. | Workers having thoracic or lumbar back pain arising from work causing an absence from work of more than 90 days, being 18 to 60 years old, having a claimed accepted for compensation by the QWCB and having the legal right to return to their job. | N= 127  TRW= 28  FR= 49  CS= 29  DEN= 21 | Questionnaire | 93.3% in the TRW group, 76.6 in the FR group, 100% in the CS group and 87.5 % in the Den group | Various | Canada | Support from leaders and co-worker support. | At 2-year follow-up, 93% of participants in the therapeutic RTW (TRW) program were working. This rate was higher than in the comparison groups. Although limited by its norm-referenced evaluation design, the results of this study indicate the importance of placing the work site in the center of the work rehabilitation process. |
| Ekberg *et al.* 2015 | Prospective Cohort | 3 months & 3-12 months. | CMDs (depression, anxiety, burnout and others) | Early and Later RTW | Demographic data, health and work ability, personal resources, work conditions and employment situation. | Sick-listed individuals with CMDs for at least 2 weeks. Aged between 18-65 years. | N= 354 | Questionnaire, Register data | 66% at baseline. | Health Care | Sweden | Personal characteristic (education, self-efficacy) and Support from leaders | Lower educational level, better work ability at baseline, positive expectations of the RTW treatment and low perceived interactional justice in interaction with supervisors were associated with early return to work. While exit behaviour or turnover intentions and need for reduced demands at work were significantly associated with a later RTW. |
| Ekbladh *et al.* 2010 | Longitudinal | 6, 12 & 24 months | MSDs, CMDs | RTW | WRI assessment items (assesses abilities and limitations, expectations of job success, take responsibility, commitment to work, work-related goals, enjoys work, pursues interest, identifies with being a worker, appraises work expectations, influence of other roles, work habits, daily routines, adapts routine to minimize difficulties, perception of work setting, perception of family and peers, perception of boss and perception of co-workers) | Workers aged 20-60 years who at one specific day in 2004 were on sick leave between 60-89 days long on at least half time. Women= 34, Men= 19, Mean age= 43 years. | N= 53 | Telephone interview | 41% | Various | Sweden | Personal characteristics (attitude-belief in self), job crafting | At all three follow-ups, results suggests that expectations of job success, taking responsibility, adapting routine to minimize difficulties and perception of family and peers are significant predictors of RTW. Overall, the WRI assessment tool contains items that could predict RTW. |
| Ekbladh *et al.* 2004 | Retrospective (longitudinal) | 2- years follow up | MSDs, CMDs | RTW | WRI assessment items (assesses abilities and limitations, expectations of job success, take responsibility, commitment to work, work-related goals, enjoys work, pursues interest, identifies with being a worker, appraises work expectations, influence of other roles, work habits, daily routines, adapts routine to minimize difficulties, perception of work setting, perception of family and peers, perception of boss and perception of co-workers) | Sick-listed workers. In the primary group, Mean age= 51 years (33-64 years range), Women= 28, Men= 20. In the secondary group, Mean age= 51, Women= 6, Men= 68 | N= 189 | Interview | 25% of the primary group and 58% of the secondary group | Various | Sweden | Personal Characteristics (Attitude, belief), Support from Leaders, co-workers | Results showed that assesses abilities and limitations, expectation of job success, taking responsibility, appraising work expectations and perception of work setting with regards to support all had predictive validity for RTW. The result emphasizes the importance of considering the unique individual’s beliefs and expectations of his or her effectiveness at work when assessing clients work ability and planning for further rehabilitation. |
| Engstrom and Janson, 2007 | Quantitative (longitudinal data) | 1, 2 & 3 year follow up | CMDs (stress) | RTW | Time being sick registered and not sick registered, Gender, employer, occupation, age, previous SA, pain diagnosis | Workers with stress-related SA with a duration exceeding 28 days. Aged between 16-60 years. Women= 76.5%, Men= 23.5% | N= 893 | SA data register | 98% | Various | Sweden | Personal characteristics (Age, length of time out of work) | Results suggest that the employer and occupational categories had minor effects on RTW after long-term SA. Furthermore, age and health related factors together with time factors seemed to be more relevant in explaining RTW. The older workforce with much poorer health who have been absent on a long-term spell are more likely to have difficulties returning to work. |
| Franche *et al.* 2007 | Prospective Cohorts | 1 & 6 months | MSDs | Relationship between RTW & SA duration | Early contact, work accommodation (offer and acceptance), HCP contacted employer, HCP advised employer on injury prevention, ergonomic assessment, RTW coordination and 20 other confounding factors | Lost-time claimants with work-related back or UE MSDs. Absent from work for a minimum of 5 days within the 14 calendar days after injury. | N= 632 | Interview, Administrative data. | 61% at baseline and 71% at 6 months follow up. | Various | Canada | Support from leaders | Findings suggest that early receipt and acceptance of a work accommodation planned and supported by the supervisor and early HCP advice to the workplace on how to prevent re-entry is associated with a shorter work absence duration measured 6 months after injury in both self-reported and administrative data. |
| Friesen *et al.* 2001 | Qualitative (Focused ethnography) | N/A | MSDs, CMDs | RTW | The worker (worker attitudes and behaviours, worker participation), Workplace system (workplace organization, trust and credibility, communication and positive relationships, workplace initiative), Health and insurer systems (communication, delays, need for education), Macro-systems themes. | Individuals able to answer the research question. Participants chosen based on knowledge, experience or importance in the work injury field and the RTW process within the work place. | N= 55 | Semi-structured interview | 100% | Various | Canada | Support from leaders and co-workers | Study revealed that delays of all types in processing or delivery of information or treatment and ineffective communication among stakeholders was perceived as barriers to RTW. While establishment of RTW programs in the workplace, effective communication and teamwork as well as trust and credibility among stakeholders facilitated RTW. The interdependence of organizational structured and human interactions was evident in successful RTW programs, which emphasized teamwork, early intervention and communication. |
| Gallagher *et al.* 1989 | Prospective | 6 months follow up | MSDs (LBP) | RTW | Illness behaviour, health locus of control, perceived stress, social support, coping mechanisms, psychiatric symptoms, work history, clinical rating. Age, length of time out of work. | Patients attending the university LBP clinic and persons who had applied to the social security administration for compensation on the basis of LBP during the same period of time, currently out of work and having worked at-least 3 months prior to their latest unemployment period. Clinic patients; age range of 22-57 years and the Social security patients; age range of 23-61 years. | N= 169 (Social security= 77, Clinic= 92) at initial assessment.  N= 150 (Social security= 63, Clinic= 87) at follow up. | Interview, self-report log, vocational questionnaire, physical examination | 88.8% at follow up. | Various | United States | Personal characteristics (age, length of time out of work) | The study identified a number of demographic, occupational and psychosocial factors that prospectively predict RTW at 6 months follow up in a sample of LBP patients. After controlling for age and length of time out of work, individual physical examination and biomechanical measures were not predictive of RTW. Exclusive reliance on the physical examination and widespread use in the determination of disability for the purpose of compensation, without consideration of psychosocial characteristics, and without adjusting for the confounding effects of age and length of time out of work are not empirically justified by the results. Data set therefore suggest that age and length of time out of work interact with psychosocial risk factors such that the strength of associations between specific risk factors and outcome depend upon the age and period of unemployment of patients. |
| Hatchard *et al.* 2012 | Qualitative | 2 Interview sessions. | CMDs | Return to mainstream work. | The worker (managing self, self-acceptance, managing lifestyle and health) The workers’ personal partnerships (relationships and partnerships, relationships form the foundation, realities challenge personal partnerships) and Workplace partnerships (relationships and demands, workplace leadership, the power of o-workers, responding to work demands) | Individuals between ages 35 and 62 who had experienced acute mental illness that had resulted in time off work and had attempted a RTW in a mainstream workplace. Women= 4, Men= 1. (age range= 35-62years) | N= 5 | In-depth semi-structured interviews | 100% | Various | Canada | Support from leaders and co-workers | Findings suggest that personal and workplace partnerships are integral to supporting workers as they take ownership of their full potential and self-direct RTW. Support from both management and co-workers were as important to promoting self-direction in the RTW process |
| Haugli *et al.* 2011 | Qualitative | N/A | MSDs, CMDs | RTW | Positive encounters, increased self-understanding, support from the surroundings. | Patients on long-term sick leave due to MSDs and/or psychological health complaints. 10 individuals who has RTW (3 Men of 46-58 years and 7 Women of 41-56 years) and 10 individuals registered with a disability pension (3 Men of 41-53 years and 7 Women of 41-56 years). | N= 20 | Semi-structured telephone Interviews | 100% | Various | Norway | Support from leaders and co-workers | The core categories describing Successful RTW include; positive encounters, an opportunity for increased self-understanding and support from the surrounding. |
| Haveraaen *et al.* 2016 | Cohort | 3 months | MSDs | RTW 3 months after RTW program. | Job demands, job control, social support, job characteristic. Other factors; age, gender, educational level, marital status, household income, diagnose, sick leave history, work status at the end of program, type of treatment, occupational sector, and physical job demands. | Workers finishing treatment at the RTW service before or during the study period, being on sick leave when they started at the RTW service and being in paid employment. Women= 76.1%, Participants with MSDs= 57.4%, multidisciplinary treatment= 60.6%, treatment from one profession= 4.3% and medical or surgical treatment= 37.1%. | N= 251 | Questionnaire, National register data | 71.1% | Health care | Norway | Support from leaders and co-workers | Results showed that having low psychological job demands, high co-worker and supervisor support and being in low strain job predicted RTW three months after end of RTW programme after adjusting for several prognostic factors. |
| Heijbel *et al.* 2006 | Prospective Cohort | 18 months follow-up | MSDs, CMDs | Prediction of RTW & RTW | sex, age, own prediction of RTW, complaints from >1 group of symptoms, duration of complaints, duration of sick leave, pain, function, physically strenuous work, contact with the workplace/workmates, perception of being welcome back to work, contact with occupational health service, contact with the regional social insurance officer, contact with the trade union and rehabilitation programme. | Persons with an ongoing spell of full-time sickness absence for 90 days or longer. Women= 484, Men= 51 | N= 535 at baseline.  N= 508 after 18 months follow up. | Questionnaire | 69% at baseline. 95% after 18 months follow up. | Various | Sweden | Personal characteristics (Attitude, age, duration of absence) | Results suggest that sick-listed person’s own positive prediction of their RTW was highly significant. Other predictive factors to RTW included being on sick leave for a period of less than 1 year, having less pain perceiving that one was welcome back to work and being under 55 years. |
| Heijbel *et al.* 2013 | Longitudinal | 2 years follow up | MSDs, CMDs | RTW | age, type of work, problems or complaints, assessment at the OHS, rehabilitation programmes, vocational rehabilitation, return to work or not after two years. | People who had reached a level of 28 days on sick leave. Women= 90% (704), Men= 10% (75), age range of 20-63 years (average age of 47 years). MSD patients= 53% (412) and Psychological/stress-related patients= 44% (340). | N= 779 | Questionnaire | 54% | Public | Sweden | Personal characteristics (age), Support from leaders. | The rehabilitation programme encountered challenges. However, counter measures were taken to facilitate coordination and communication. People with MSDs often received both multimodal and vocational rehabilitation. Vocational rehabilitation was advocated for people who were under 55 years of age, and for those with stress-related problems. The strongest predictive factors for RTW were; having received only vocational rehabilitation and being under 45 years of age. The study shows the need for coordination between multiple stakeholders. It suggests that supervisors should pay attention to people who have MSDs and are older as soon as the problem emerges. |
| Hoefsmit *et al.* 2014 | Qualitative | N/A | MSDs, CMDs | RTW | Environmental factors (social support, belief that RTW supports health, adequate cooperation between stakeholders, work supervisor’s communication skills) and personal factors (employee’s positive perception of the situation). | Employees who had been absent for more than 42 days and less than 2 years or had experienced long-term sick leave and had resumed work less than one year before the interview, employers who represented the organisation’s RTW policy and supported absent employees to resume work and Ops who supported individual employees on sickness absence to RTW. Men= 20, Women= 14 | N= 34 | Open-ended Interviews | 100% | Various | Netherlands | Support from leaders, Personal Characteristics (Attitude) | Results showed that both environmental (social support from relatives, belief that work stimulates health, adequate co-operation between stakeholders in RTW; E.G employees, employers and Ops, and the employer’s communication skills) and personal factor (positive perception of working condition) stimulated RTW. Most factors stimulated RTW directly. In addition, adequate treatment and social support stimulated medical recovery. |
| Hu *et al.* 2014 | Prospective Cohort | 0.5 & 8 months follow up | MSDs | RTW & SA duration | Same company as before, same job title as before, with signed job contracts, receiving work-related injury insurance, monthly salary of RTW versus pre-injury, work duration per week(hours), satisfaction with RTW, way of achieving RTW. Other potential predictors; demographic, clinical and socio-economic. | Workers with work-related hand injury. 55 years and younger for women, 60 and younger for men. Median age= 33.0 years, Median work experience= 1.4 years, Males= over 80% and from rural areas, married= 69.5%, middle school education= 54% | N= 246 | Structured Questionnaire via telephone (interview) | 96% | Various | China | Support from leaders | During the 8-month follow up, 78.1% (192 cases) returned to work successfully with a median absence duration of 44 days. Study indicated that multi-dimensional factors were significant in determining RTW. Factors from demographic, clinical, economic and psychological domains affected RTW in the univariate analyses. Receiving timely treatment, less serious injury, no tendon trauma and no skin loss were found to be significantly beneficial to RTW, while workers with decreased monthly salary during absence and lower pre-injury salary are likely to take longer sick leave. Most of the workers successfully achieved RTW after work-related hand injury. Proper clinical treatment and post-injury rehabilitation as well as economic and social support seem to have played a vital role in prompting RTW that should be prioritised for intervention strategy. |
| Huijs *et al.* 2012 | Prospective Cohort | 2- year follow up | MSDs, CMDs | Duration until full RTW. | Gender, age, marital status, working hours, children living at home, education, ethnicity, contract type, working status, depression, anxiety, coping (active-problem-solving), coping (avoidance), RTW self-efficacy, expectations work environment, physical exertion, level of RTW, days until full RTW. | Employees sick-listed for 19 weeks. Mean age= 46.6 years, Women= 58.4%, 60% older than 45 years. Employment contact= 31.7h. | N= 682 | Questionnaire | 52% | Various | Netherlands | Personal Characteristics (age, educational level, self-efficacy, job contract) | Result showed that reporting both physical and mental problems as reason for sick leave was associated with a longer duration until full RTW. Non-parametric cox survival analysis showed that partial return to work at baseline and a lower age predicted full RTW. For employees with physical conditions, high level of education and RTW self-efficacy predicted RTW. For employees with mental complaints, those with permanent job contract returned fully while those with both physical and mental complaints were associated with longer duration until full RTW. |
| Janssen *et al.* 2003 | Prospective Cohort | Every 4 months for a period of 3 years (For questionnaires), 2 months after ill-health, a follow up every 2 months and a final follow up 1 year after reporting ill (for interviews) | MSDs, CMDs | Not working, RTW with adjustments & Full RTW. | Demographic covariates (gender, age), DCS variables (psychological job demands, supervisor support, co-worker support, decision latitude; skill discretion & decision authority) | Employees sick-listed for 6-8 weeks. | N= 455 | Questionnaire, Interviews | 87.5% at T2 | Various | Netherlands | Support from Leaders | Results indicated that high job demands were the least predictive of full RTW. However, the likelihood of employees with high job demands returning to work with adjustments was higher than the likelihood of them not working. Therefore, job demands might also work as a pressure to RTW. Furthermore, high skill discretion in combination with high job demands predicted working with adjustments in comparison with not working. High supervisory support was the most predictive of RTW without adjustments and the least predictive of not working. |
| Jakobsen and Lillefejell, 2014 | Qualitative | N/A | MSDs | Successful RTW | Employees’ experiences of factors affecting the RTW process (mobilizing personal resources (job crafting practices), balanced daily life, needed dialogue and social support) and Factors in the employers’ experiences as important for a successful RTW (adjustment at work, desired to be more actively involved in the RTW process and gap between employment and reality). | Long-term sick listed employees with chronic musculoskeletal pains who participated in the rehabilitation programme at the rehabilitation centre and had all returned to the same job, full or part time. Have national insurance benefit in the form of sickness benefit or rehabilitation benefit in 3 months or more. Men= 2, Women= 4. Aged 40-57 years. | N=6 | Interviews | 100% | *****Various | Norway | Job Crafting, Support from leaders and co-workers | Results suggest that successful RTW to work was dependent on employee’s ability to identify and mobilize their personal resources, adapt a balanced daily life, require a positive dialogue with family, colleagues and their employer, while employers underlined the need for a helpful adjustment at work and how they wanted to become more involved in the rehabilitation process. |
| Jensen *et al.* 2012 | Randomized Clinical Trial | 2 year follow up | MSDs (LBP) | Sustainable RTW | RTW and weeks on sick leave | Participants on sick leave for 3-16 weeks due to LBP, 16-60 years of age, and able to read and speak Danish. | N= 351 (Multidisciplinary intervention=176 , Brief intervention=175 )  N= 344 after follow up ( Multidisciplinary intervention=124 , Brief intervention=120) | Questionnaire | Multidisciplinary intervention group = 70.5%, Brief intervention group= 68.6% | Various | Denmark | Support from leaders | During the 2 year follow up, 80.0% and 77.3% had RTW for at least four weeks continuously, and the percentages with RTW at the 104^th^ week were 61.1% and 58.0% in the brief and multidisciplinary intervention groups respectively. At the 104^th^ week, 16.6% and 18.8% were on sick leave in the two groups, respectively, and 12% were employed in modified jobs or participated in job training. The number of weeks on sick leave in the first year was significantly lower in the brief intervention group than in the multidisciplinary group, but during the second year, the number of sick leave were not significantly different between the intervention groups. Subgroups characterised by specific work related factors modified the effect of the intervention groups on RTW rates. No difference in sick leave relapse was found between the intervention groups. The effects of the brief and multidisciplinary interventions at the two-year follow up were in general similar to the effects at the one-year follow up. |
| Johansson *et al.* 2006 | Cross-Sectional | 1 year | CMDs | RTW | Adjustment latitude, age, health, stimulating work, demanding household work. | Salaried employees who had been on sick leave for at least 90 days for one of 16 diagnoses in 2000.Age range= 21-66 years, Women= 1783, Men= 1273. | N= 3056 | Questionnaire | 54.7% | Private | Sweden | Job Crafting | Among women 32% were fully back to work, 34% were partly back and 34% were still on sick leave. Comparable figures for men were 33%, 32% AND 36%. For both men and women the likelihood of RTW increased with increasing opportunity to adjust their work. Adjustment latitude thus increased returning to part-time as well as full-time work. |
| Karlson *et al.* 2010 | Prospective controlled trial | 1.5 years follow up | CMDs | Successful RTW | Patient-supervisor communication. Age and Gender. | Employment sick listing at least half time for 2-6 months from a previously healthy state, and having an International Classification of Diseases (ICD-10) diagnosis within the F43 category (reaction to severe stress, and adjustment disorders, except post-traumatic stress disorder (F43.1), due to predominantly work-related stressors. Women= 59 (intervention group) and 56 (control group), Mean age= 46.6 and 46.1 years in both intervention and control group respectively. | Intervention group; N= 74, Control group; N= 74 | Questionnaire, Interviews | - | Various | Sweden | Support from leaders | There was a linear increase of RTW in the intervention group during the 1.5-year follow up, and 89% of subjects had returned to work to some extent at the end of the follow up period. The increase in RTW in the control group came to a halt after 6 months, and only 73% had returned to work to some extent at the end of the 1.5-year follow up. Results suggest that workplace-oriented interventions involving dialogue with supervisors are effective in improving long-term RTW for patients on long-term sick leave due to burnout. |
| Karlson *et al.* 2014 | Prospective Controlled | 1.5 years originally, and then after 1 years. | CMDs (Burnout) | Long-term stability of RTW | Patient-supervisor communication. Age and Gender. | Consecutive new sick-listed cases for the period 2003-2006. Those in employment, sick-listed for at-least half time for 2-6 months following a previously healthy state. Women= 81%, Mean age= 45.5 years of range 25-62 years. | N= 148  (Intervention group= 74, Control group= 74) | Questionnaire, Interview and Team supported dialogue. | 86% | Various | Sweden | Support from leaders and Personal Characteristics (age) | Test over all 130 weeks showed a GROUP*WEEKS interaction effect, indicating differential group developments in RTW, though similarly high at week 130 in both groups with 82.4% of the intervention group and 77.9% of the control group having RTW. A significant interaction with age led to separate analyses of the younger and older subgroups, indicating a stable pattern of superior RTW only among younger participants in the intervention group. Results indicated that workplace-oriented interventions involving both supervisors and employees showed long-term stability on RTW only among younger participants. |
| Krause *et al.* (2001) | Retrospective Cohort | 1-4 years follow up | MSDs (LBP) | Time to RTW | Psychosocial job factors, duration of disability, injury history and severity, physical workload and demographic and employment factors. | A complete 3-year cohort of 850 compensated low back injury cases drawn from all workers administered at three district offices of a large worker’s compensation insurance carrier. An ICD-9 code indicative of a definite LBP diagnosis on any medical bill record of the first physician visit or on any bill record of a physician visit within 14 days after date of injury, within 14 days after the first physician visit and within 90 days after the date of injury. Acute phase= Mean age= 37.3 years old, Female= 30%, Male= 70%. Sub-acute/chronic phase= Mean age= 38.6 years old, Female= 32.2%, Male= 67.8%. | N= 721 at telephone follow up.  N= 433 at interview. | Interview, survey | 60% at point of interview | Various | United States | Personal characteristics (job control; control over work and rest periods) and Job Crafting | High physical and psychological job demands and low supervisory support are each associated with about 20% lower RTW rates during all disability phases. High job control, especially control over work and rest periods were associated with over 30% higher RTW rates, but only during the sub-acute/chronic disability phase starting 30 days after injury. Job satisfaction and co-worker support were unrelated to time to RTW. |
| Labriola *et al.* (2006) | Cohort | 1 year follow up | MSDs (wrist pain) | RTW | Psychosocial work environment risk factors (psychologic demands, decision authority, skill discretion, meaning of work and predictability of work, co-worker social support and supervisory social support), Physical work environment risk factors (stooping work position, twisting the back, lifting more than 30kg, pushing/pulling heavy burdens, full body vibration and repeating the same job task many times per hour). Health behaviour, body mass index and general health | Employees who experienced SA periods exceeding 2 weeks during 2 years of follow up. | N= 428 | Questionnaire and Register data. | 75.6% | Various | Denmark | Personal characteristics (psychologic demands, decision authority, skill discretion, meaning of work and predictability of work), support from leaders and co-workers. | Of the 428 employees who were sick-listed for more than 2 weeks, 367 returned to work within 1 year after onset of SA, while 186 returned to work within 4 weeks. At the individual level, significant associations were found between one psychosocial (low meaning of work) and four physical factors (stooping or twisting the back, lifting more than 30kg, and reporting repetitive job tasks) and RTW within 4 weeks. The association was a decreased chance of RTW. While within 1 year, only 2 physical factors (being exposed to stooping work position and having repetitive job task) decreased the chance of RTW. |
| Lagerveld *et al.* 2010 | Longitudinal | 3 waves. Baseline, 3 & 6 months | CMDs | RTW | Self-efficacy, depression, locus of control, coping and physical workload | Sample 1- Employees sick-listed for 13 weeks. Average age of 46 years, Females= 54% and worked for an average of 32 hours per week.  Sample 2- Employees sick-listed due to CMDs and are going to receive psychotherapy shortly after baseline measurement. Average age of 41 years, Female= 57% and worked an average of 33 hours per week.  Sample 3- Employees on sick leave and have had contact with their occupational Physician during the inclusion period. Average age of 44 years, Women= 47% and working an average of 33 hours per week. | N= 2214 (Sample 1= 1934, Sample 2= 189 and Sample 3= 91) | Questionnaire, Files of the occupational health organization. | 36% in sample 2 and 21 % in sample 3 | Various | Netherlands | Personal characteristics (self-efficacy) | The associations with general self-efficacy, locus of control, coping, physical workload and mental health problems support the construct validity of the scale. Most importantly, results indicated that RTW self-efficacy proved to be a robust predictor of actual RTW within three months. |
| Laisne *et al.* 2013 | Prospective Cohort | Baseline, after 2 & 8 months follow-ups. | MSDs | RTW | Age, gender, duration of symptoms, pain severity, disability, work importance, work support, work satisfaction, recovery expectations, depression, anxiety, global distress severity index, post traumatic symptoms and readiness to change. | Working-age individuals suffering from musculoskeletal disorders and receiving compensation benefits. Those whose Musculoskeletal injuries resulted from a fall, an impact or repetitive strains, and comprised or severed relationship to employer. Age between 18-55 years old. Men= 47, Mean age= 37.73 years, average of 11.01 years of education and $27,431 of pre-injury income per year. | N= 62 | Questionnaires, administrative database | 34.4% | Health and safety | Canada | Personal characteristics (age, gender), Support from co-workers | Multivariate analysis indicated that at 2 months, gender, work recovery expectations and importance of work were predictive of work outcomes. While at 8 months, age, medical consolidation, trauma symptoms, work support and importance of work were predictive of work outcomes. |
| Lammerts *et al.* 2016 | Cohort (Longitudinal data) | Baseline, 2 & 4 years follow up. | CMDs (depression & anxiety) | Sustainable RTW in 2 years | Demographic Characteristics (sex, age, partner status, education and net income), Personality Characteristics (neuroticism, extraversion, openness, agreeableness, conscientiousness and locus of control), Disorder-Related Characteristics (diagnosis anxiety or depression, severity depression, severity anxiety, percentage of time depressive symptoms, percentage of time anxiety symptoms, use of anti-depressants, specialized mental health care) and Work-Related Characteristics (employment status, SA, job demands, decision authority, skill discretion, social support, job security and type of worker). | Participants with long-term depressive and anxiety disorders. Ages 18-65 years old. Female= 66.5%, Mean age= 42.32 years | N= 215 (T0= 176, T1= 39) | Data from the Netherlands study of depression and anxiety (NESDA) | 81.8% at T0 and 22.3% at T1 | Various | Netherland | Personal characteristics (age,) | Results shows that In 2 years, 51.6% of participants returned to work sustainably and age, household, income, extraversions, employment status, skill discretion and job security were significantly associated with sustainable RTW in 2 years in the univariate analysis. While the multivariate analysis revealed significant associations between sustainable RTW and age, household ad being on sickness benefit versus being employed. |
| Lederer *et al.* 2012 | Cohort (longitudinal data) | 5 years follow up | MSDs | Time to RTW following long-term disability | Age, number of dependents, gross annual income, perceived economic status, occupational category, perceived physical workload, hours of paid work per week, job satisfaction, work experience, job seniority, union membership, employment status, company size, score of job insecurity, awareness of OHS program in the workplace, injury site, nature of MSD, claim history | Adults on long-term disability due to work-related MSDs of the back, neck or upper limbs receiving compensation benefits for at-least 2 months at study entry. Age range= 18-55 years old. Men= 286, Women= 169 | N= 455 | Structured interviews and administrative databases. | 100% | Various | Canada | Personal characteristics (age, gender, economic status, annual income, job contract) | Time to RTW for both men and women on long-term disability were similar, but many personal and occupational factors influencing RTW differed by gender. Women’s risk factor included older age, poor to very poor perceived economic status, working ≥40h/week and having dependents and awareness of workplace-based occupational health and safety program. In men, being over 55 years old, poor perceived economic status, working ≥40h/week and high-perceived physical workload and higher job insecurity negatively influenced time to RTW. In both men and women, probabilities of not returning to work varied widely according to worker’s specific profile of personal and occupational factors. |
| Loisel *et al.* 1997 | Randomized Clinical Trial | 1 year follow up | MSDs (back pain) | RTW | Duration of absence from work, functional status, pain level, minor comorbid diseases. | Workers with thoracic and lumbar back pain incurred at work that had caused an absence from work for more than 4 weeks and less than 3 months, age from 18-65 years, and back pain accepted for compensation by the Quebec WCB. | N= 104 (Usual care= 26, Clinical= 31, Occupational= 22, Full intervention= 25) | Questionnaire | - | Various | Canada | Support from leaders | The full intervention group returned to regular work 2.41 times faster than the usual care intervention group. The specific effect of the occupational intervention accounted for the most important part of this result, with a rate of return to regular work of 1.91. Pain and disability scales demonstrated either a statistically significant reduction or a trend toward reduction in the three intervention groups, compared with the trend in the usual care intervention group. |
| Lydell *et al.* 2009 | Prospective and comparative Follow-up | 1 year, 5 and 10 years follow up. | MSDs | Sustainable RTW | Gender, age, marital status, spouse disability pension, education, socio-economic division, diagnosis and working situation. | Working-age people aged 18-65 years, who were sick-listed due to MSDs. | N= 385 at baseline and 1 year follow up. N= 243 at 5 and 10 years follow up. N= 183 (working full-time group= 110 and sick-listed group= 73) final inclusion. | Questionnaire | 69% at 5 and 10 years follow up. | Various | Sweden | Personal characteristics (period of absence, age, gender, educational level) | Results indicated that the number of sick-listed days before rehabilitation, age, self-rated pain, life events, gender, physical capacity, self-rated functional capacity, educational and light physical labour were predictors of long-term RTW. |
| Lysaght and Larmour-Trode, 2008 | Qualitative | - | MSDs, CMDs | RTW | Support in the workplace; emotional, information, instrumental and appraisal support | Workers and supervisors who had experienced or supervised work re-entry event within the previous 12 months. Those who had experienced workplace injury or disability within the previous 12 months and returned to work on modified duties or with modified equipment or other supports. Workers; Females= 14, Males= 4. Average age= 47.7 years (range= 24-61 years). Supervisors: Females= 2, Males= 6. Average age= 44.6 years (range= 37-53 years). | N= 26 ( Supervisors= 8, Previously Injured workers= 18) | Interviews | 100% | Various | Canada | Support from leaders and co-workers | A full range of social dimensions were reported to be relevant, and were arising from a variety of sources (e.g. supervisors, co-workers, disability manager, work unit and outside of work). Respondents identified trust, communication and knowledge of disability as key precursors to a successful RTW process. |
| Marhold *et al.* 2001 | Randomized controlled | Pre-treatment, post treatment, 4 & 6 months follow up. | MSDs | RTW. Reduction in SA. | Number of days of sick leave. Well-established self-reported inventories like; Multi-dimensional pain inventory (MPI), Coping strategies questionnaire (CSQ), Beck depression inventory (BDI), Pain and impairment rating scale (PAIRS) and Disability rating index (DRI). | Women between 25-60 years old, a diagnosis of MSDs, no psychotic illness, no planned operations and being gainfully employed. Mean age= 46 years. | N= 72 (long-term sick leave of >12 months= 36, Short-term sick leave of 2-6 months= 36) | Questionnaires, beck depression inventory, disability rating index, pain & impairment rating scale | 91.7% | Various | Sweden | Job crafting | Results showed that cognitive-behavioural RTW program was more effective than treatment as usual in reducing the number of days on sick leave for patients on short-term sick leave. The treatment program also helped the patients on short-term sick leave to increase their ability to control and decrease pain and to increase general activity level compared to the control condition. |
| Martin *et al.* 2015 | Mixed study | 2 years after first interview for individual interviews.  2 years 2 months after first interview for group interviews.  Multidisciplinary team observed on 4 occasions. | CMDs | Early RTW and reduced SA. | Recruitment and reach, multidisciplinary rehabilitation activities, coordination of stakeholders, cooperation with SIOs, participant satisfaction and context. | Employees aged between 20 and 60 years, on SA of 4-12 weeks duration because of CMDs such as depression, anxiety or stress-related conditions. Women= 142, Men= 71 | N= 213 | Individual and group interviews, observations, national registers and documents from the intervention. | 83.5% | Various | Denmark | Personal characteristics (Positive expectations), Support from leaders. | The quality of the implementation varied greatly across the three settings. Barriers included lack of skills to assess MHPs according to the inclusion criteria, different interpretations of SA legislation among stakeholders, competing rehabilitation alternatives, and lack of managerial support for the intervention. An important facilitator was the motivation and availability of resources to solve disagreements through extensive communication. |
| Muijzer *et al.* 2011 | Case Report | - | MSDs, CMDs | RTW & RTW Effort sufficiency. | Personal (age, gender, education, reason of absence, tenure, periods of complete disability, periods of work resumption) and external factors (SA work related, relationship employer/employee and conflict). | Sick-listed Employees who have not returned to work fully and are not receiving the original level of income and are not fully disabled. Average age= 47 years. Male= 180 (43%), Female= 235, low education level= 20%, medium educational level= 60% and high educational level= 20% | N= 415 | Close-ended Questionnaire | - | Unknown | Netherlands | Support from leaders, Personal characteristics (Educational level) | Using the multiple logistic regression analysis, the only factor related to RTW effort sufficiency was good employer-employee relationship. Factors related to RTW were high education, no previous periods of complete disability and a good employer-employee relationship. |
| Nielsen *et al.* 2010 | Prospective follow up (Longitudinal) | 52 weeks follow up | CMDs (stress, burnout, depression, anxiety) | Time to RTW | Gender, age, RTW expectancy, prior absence with MHP, occupation, self-reported reason for absence. | Employee absent due to MHP, employees who reported somatic complaints. Sickness absence not more than 12 weeks. Male= 190, Female= 454, Age range of 19- ≥50 years, Mean age= 40 years | N= 644 | National register for social transfer payments, Questionnaire | 100% | Various | Denmark | Personal characteristics (Positive attitude) | Employees sick-listed with self-reported stress/burnout returned to work faster than those with self-reported depression and other MHPs do. A positive RTW expectancy of the sick-listed person and no prior absence with HPs were associated with a shorter time to RTW. |
| Nielsen *et al.* 2013 | Mixed | Baseline and 6 months follow up. | CMDs (depression, anxiety, stress) | RTW | Age, educational level, workplace, size of workplace, RTW status, employment status, major depressive inventory symptom score | Employees sick-listed due to CMD and had applied for sickness benefit compensation. Male= 44, Female= 182. Age range of 19- 50 years and older. | N= 226 | Questionnaires register and interviews. | 41% at baseline. 76% at 6 months follow up. | Various | Denmark | Support from leaders and co-workers | High support was most often reported from the personal and health system, while encounters with social insurance officers were least often reported to be highly supportive. Colleagues were more often reported to be highly supportive (49%) than supervisors (30%).Gender differences remained statistically significant in both contact and encounter assessments. Women considered their supervisors as less supportive, while their friends were highly supportive. |
| Nieuwenhuijsen *et al.* 2004 | Longitudinal Cohort | Baseline, 3month, 6 months and after 1 year. | CMDs | Time to RTW | Communication with employees, promoting gradual RTW and consulting other professionals. | Employees on sick leave due to MHPs for less than 6 weeks. Mean age= 44.2%, Male= 42% | Supervisor: N= 85. Employees; N=198 | Questionnaire, Telephone Interview | 94% at baseline | Health care | Netherlands | Support from leaders | Better communication between supervisor and employee was associated with time to full RTW in non-depressed employees. For employees with a high level of depressive symptoms, this association could not be established. Consulting other professionals was more often associated with a longer duration of sickness absence for both full and partial RTW. If sickness absence had financial consequences for the department, the supervisor was more likely to communicate frequently with the employee. In conclusion, supervisors should communicate more frequently with employees during SA as well as hold follow up meetings more often as this is associated with a faster RTW in those employees. |
| Opsahl *et al.* 2016 | Randomized controlled trial | 12 months follow up | MSDs (LBP) | Actual RTW | Age, gender, education, covariates, co-worker social support, job satisfaction, and return to work expectancies. | Employees on sick leave due to LBP for 2-10 months. At least 50% sick-listed, at least 50% employed, age range= 20-60 years, Men= 49.7%, Mean age= 44.3 years old. | N= 574  (Intervention group= 414 and Control group= 160) | Questionnaire | 98.8% | Various | Norway | Personal characteristics (high RTW expectancies, gender) | Regardless of gender, high expectancies of returning to work were a strong and significant predictor of RTW at 12 months. While high job satisfaction were not a significant predictor. There were no differences in the levels of expectancies or overall job satisfaction between men and women. However, men had in general higher odds of returning to work compared to women. |
| Post *et al.* 2005 | Longitudinal | 10 months follow up | MSDs, CMDs (stress) | RTW | Duration of employment in present job, total duration of employment, extent of employment, status of employment, type of working hours, management position, vocational sector, and industry. | Employees on sick leave for a maximum of 12 weeks. Men= 466, Women= 460, age range= 18-63 years (Mean age= 46 years), very low education= 8%, low education= 33%, medium education= 30%, high education= 30% | N=926 | Questionnaires | 86% | Various | Netherlands | Support from leaders and co-workers | The multivariate model showed that working in one of the vocational sectors public administration, construction, financial and commercial services, transport or education and having a low co-worker support was related to longer duration of RTW. While having a low supervisory support was associated with a higher rate of RTW. |
| Poulsen *et al.* 2014 | Randomized controlled trial | 52 weeks follow-up | CMDs | Recovery from SA | Age, gender, education, employment status, purchase of prescribed medicine, contact with own general practitioner, and history of hospital admission. | 3 Municipalities that had separated sub-units of their sickness benefit management offices serving the same population allowing for randomization at the individual level of sick-listed beneficiaries to an intervention or control office; The sickness benefit office sub-units were geographically separated, thereby reducing the risk of intervention spill-over between CTM intervention and ordinary sickness benefit management; and the number of sick-listed beneficiaries eligible for the study was sufficiently high to generate a large intervention and comparable control group. Participants sick-listed for 8 weeks. | N= 3105  (Intervention group= 1948 and Control group= 1157) | Questionnaire, interviews | - | Various | Denmark | Support from leaders | The intervention effect differed significantly between the municipalities. In one municipality, the intervention resulted in a statistically significant increased rate of recovery from long-term SA. In the other two municipalities, the intervention did not show a statistically significant effect. Adjustments for a series of possible confounders only marginally altered the estimated hazard ratio. The effect of the intervention differed substantially between the three municipalities, indicating that the contextual factors are of major importance for success or failure of this complex intervention. |
| Reiso *et al.* 2003 | Follow-up | 2 year follow-up | MSDs (Back) | Time until RTW | Age, gender, diagnoses, pain intensity, work ability, self-predicted absence status, RTW. | Patients certified as sick who attended a back disorder outpatient clinic from September 1997 to December 1998. Age range= 20-63 years, Men= 65% | N= 190 | Questionnaire | - | Various | Norway | Personal characteristics (age, diagnosis, self-assessed work ability, self-prediction) | According to the multiple cox regression analysis, age of 40 to 49 years, high pain intensity, low self-assessed work ability and a self-predicted absence status of not returning to work predicted longer time until RTW. Back disorders with radiation predicted shorter time until return to work. The CCP/WONCA chart’s physical fitness, daily activities, overall health and change in health were associated with time until return to work in the univariate analyses only, as was the duration of sickness certification episodes from start to inclusion and the degree of sickness certification at inclusion. In conclusion, information about the age of patients, diagnoses, pain intensity, self-assessed work ability and self-predicted absence status may be used as predictors of time until RTW in patients with back disorders. |
| Roelen *et al.* 2012 | Mixed | 1.5, 3, 6, 12 & 12 months | CMDs (emotional, neurotic, somatoform, stress, mood disorder (depression)) | RTW | Age, gender and socioeconomic position. | Employees on SA due to mental disorder. Men= 21,146, Women= 30,608, <35years- ≥55 years | N= 51,754 | - SA Register | - | Various | Netherlands | Personal characteristics (age, gender, socioeconomic position) | Employees with emotional disturbances had the highest RTW rates; 95% and 98% after 1 and 2 years, compared to 89% and 96% of employees with neurotic, somatoform and stress-related disorders and 70% and 86% of employees with mood disorders respectively. Women resumed their work later than men. While younger employees with emotional disturbances, neurotic, somatoform and stress-related disorders had earlier RTW than older employees and employees with low socioeconomic position had earlier RTW than those with high socioeconomic positions. RTW rates and probabilities differed across categories of mental disorders. Age and socioeconomic position were associated with RTW of employees with emotional, neurotic, somatoform and stress-related disorders but not among those experiencing mood disorders. |
| Selander *et al.* 2015 | Mixed (Explorative method/descriptive design) | April 2012, 2^nd^ Reminder and June 2012. | MSDs, CMDs | RTW | Employee’s contact with workplace actors. | Sick-listed individuals on full sickness absence of between 60-90 days and permanently employed. Age range 16-65 years old. Men= 215, Women= 316, Mean age= 51.7% and 50.3% for men and women respectively. | N= 1112 initial selection.  N= 390 in April 2012. N= 502 on second reminder and N= 534 in June 2012. Total respondent= 531 | Questionnaire | 35% at first dispatch. 45% at 2^nd^ reminder. 48% at final reminder. | Various | Sweden | Support from leaders and co-workers, Personal characteristics (positive attitude) | Results showed that employees had frequent and in most cases, appreciated contact with their supervisors and co-workers. Contact with other workplace actors; that is, the occupational health unit, the union representative and the human resources department, were less frequent. Employees who experienced the contact as supportive and constructive were far more positive and optimistic than others regarding RTW. |
| Shaw *et al.* 2008 | Case Study | - | MSDs (shoulder) | Number of days to RTW | Workplace-based RTW program. Examination of the management of shoulder injuries at work. | Workers who were diagnosed with rotator cuff injuries from January 1999 to December 2003. Age range= 18-45 years old | N= 184 | Telephone and in person in-depth Interview, Onsite visits, Document review | 100% | Manufacturing | Canada | Support from leaders | Findings revealed that workplace-based RTW programs were consistent with and shaped by the organizational culture of problem solving, knowledge exchange and equitable participation of workers, supervisors and health professionals. These components contributed to the problem achieving the following outcomes; one-third of workers were placed on modified duties within 3days, 56% of workers who engaged in an early RTW program returned to work within one month. Overall, 87.8% of workers with rotator cuff injuries successfully returned to pre-injury work. |
| Shiri *et al.* 2011 | Randomized Controlled Trial | 2, 8, 12 and 52 weeks follow up. | MSDs | Reduction of SA | Pain intensity, pain interference with work, leisure time and sleep. Age, physical activity, lifting, arm elevators, forceful or pinch grip, job strain, fear avoidance. | Subjects seeking medical advice due to upper extremity symptoms whose symptoms or the exacerbation of symptoms had started less than 30 days prior to the medical consultation and immediate sick leave was not required. Age range= 18-60 years. | N= 177 (Intervention= 91, Control = 86) | Interview, internet and mailed questionnaire and administrative data. | At baseline, 98% in both intervention and control groups. At 52 weeks follow up, 71% in the intervention group and 75% in the control group. | Health care | Finland | Support from leaders | During the first three months of follow up, the percentage of employees with SA due to UE or other MSDs did not differ between the intervention and control group, but the total number of SA days In the intervention group was about half of that in the control group. During 4-12 months of follow up, the percentage of employees with sickness absence due to upper extremity disorder or upper extremity and other MSDs combined was lower in the intervention group than the control group. (Where intervention involved participation of supervisors.) Results suggest that early ergonomic intervention reduces SA due to UE or other MSDs. |
| Stahl and Stiwne, 2014 | Qualitative | 2 Occasions (interviewed between 2005 and 2006 and between 2008 and 2009) and a follow up after 4 years. | CMDs | RTW | Restitutive and Contingent Narrative (Possibility of accommodation and support from employers, colleagues, healthcare professionals and insurance officials and quality of interactions) | Persons sick-listed with CMDs and on sick leave. Women= 7, Men= 1, Age ranged= 30 and 57 | N= 8 | Interview | 100% | Various | Sweden | Support from leaders and co-workers | In the restitutive narratives, RTW was considered as essential for returning to life as it was, and support from managers and colleagues facilitated a successful return. |
| Steenstra *et al.* 2006 | Randomized controlled trial | 12, 26 & 52 weeks follow-up. | MSDs (LBP) | Lasting RTW | Pain intensity, functional status, quality of life and general health. | Workers sick-listed for a period of 6 weeks due to LBP. Age range= 18-65 years. | N= 196 (Workplace intervention= 96 and Usual care= 100) | Questionnaire | 100% | Health care | Netherlands | Support from leaders | The workplace intervention group returned to work 30.0days earlier on average than the Usual care group at slightly higher direct costs. Workers in the clinical intervention group that had received usual care in the first 8 weeks returned to work 21.3 days later on average. The group that had received the workplace intervention in the first 8 weeks and the clinical intervention after 8 weeks returned to work 50.9 days later on average. A workplace intervention was more effective than the usual care in RTW at slightly higher costs and was equally effective as usual care at the equal costs on other outcomes. A workplace intervention thus results in a safe and faster RTW than usual care at reasonable costs for workers on sick-leave for two to six weeks due to LBP. |
| Steenstra *et al.* 2009 | Exploratory sub-group analysis in a Randomized controlled trial | 2, 6, 8, 12, 26 & 52 weeks follow up | MSDs (LBP) | Lasting RTW | Workplace intervention (graded activity) and Usual care (Age, sick leave in previous 12 months, female, pain, functional status and heavy work). | Workers with LBP on sick leave last 2-6 weeks. Age= 18-65 years. Women= 57.1% | N= 196 (Intervention= 96, usual care= 100) | - Questionnaire, visual analogue scale, Roland Morris disability scale, self-report. | - | Various | Netherlands | Personal characteristics (age, medical history and previous sick-leave) | The interaction between age and the workplace intervention indicates a modifying effect. The workplace intervention was effective for RTW only for older workers (44 years and above) and workers with previous sick leave in the last 12 months. The interaction between sick leave in the previous 12 months and the workplace intervention is significant. A modifying effect of gender, heavy work and pain score and functional status on the effectiveness of this intervention was not found. |
| Stoltenberg *et al.* 2010 | Longitudinal | 2-3 years follow-up | MSDs, CMDs | RTW | Gender, age, primary diagnosis, municipality, ethnicity and income | Social workers in six municipalities in East Denmark sick-listed on a long-term basis from 1 October 2002 to 31 December 2005. Age range= 18-58, Mean age= 42.5 years. Men= 3139, Women= 4641. | N= 7780 at baseline.  N= 5562 at 3 years. | Dream Register, | 71.5% at 3 years | Health care | Denmark | Personal characteristics (age) | After 1.5 years, 55.2% of the population had returned to work and this level was maintained through the remaining follow-up period. All the included potential determinants were found to be significantly related to RTW at 1 and 3 years. The effects of sex, ethnicity, and income were found to be nearly constant over time. The effects of municipality, diagnosis and age changed markedly over time and mostly during the first year. |
| Tenhiala *et al.* 2013 | Prospective study | Initial survey in 2004. SA records tracked between 2005 and 2006. | CMDs | SA | Perceptions of organizational justice, SA, age, job demands, gender, tenure in current work position, occupational group, work unit, job demands, health behaviours | Employees on SA. Women= 81%, Served in non-communal occupations= 83%, Mean age= 46.2%, Age range= <35 - >55 years old. | N= 37,324 | Questionnaire, SA records | 66% in 2004. | Health care | Finland | Support from Leaders | Results suggest that age moderates the association between perceptions of procedural justice and long SAs after controlling for gender, tenure, occupational group, work unit, job demands and health behaviours. When older employees experienced a high level of procedural justice, they were less likely to take short, non-certified SAs from work. Finally, results suggest that high quality relationships with supervisors can prevent both short and long spells of sickness absence at all ages. |
| Tjulin *et al.* 2011 | Qualitative | - | MSDs, CMDs | RTW | Policies and organizational structure for RTW, Social demands & expectations and supervisory management of RTW. | Workers, co-workers, human resource manager and supervisors across 7 units in 3 municipalities. Work units that had experienced a recent RTW of a sick-listed worker who had been on sick-leave for at-least 1 month and when the re-entry of the sick-listed worker did not occur more than 3 months before the interview date. | N= 33 | Interview | 100% | Public | Sweden | Support from Leaders and Co-workers. | Key findings that emerged during analysis showed that some co-workers have a more work-task oriented approach towards return to work process, whilst others had a more social relational approach. In both situations, the social relations worked hand in hand with job tasks (how task were allocated and how returning workers were supported by others) and could make or break the RTW process. The constant communication amongst the co-workers and between the co-workers and supervisors and the re-entry workers and updates on the return to work process facilitated an understanding among co-workers about the situation of returning worker appeared to facilitate RTW. |
| Van Beurden *et al.* 2015 | Cluster randomized controlled trial | Baseline & 3 months follow up. | CMDs | RTW | RTW self-efficacy, RTW, personal, health-related and work-related variables. | Occupational physicians. Workers on sick leave due to a MHP. Age range= 18-64 years. Women= 60%, highly educated= 2/3, mean number of contracted hours= 32h a week | N= 66 (occupational health physicians; 32 in the intervention group and 34 in the control group)  N= 128 (sick-listed workers) | Structured telephone questionnaire, questionnaire. | For workers; 93% at baseline and 95% at 3 months follow up. | Health care | Netherlands | Support from leaders, Personal characteristics (self-efficacy) | 28.9% of workers fully returned to their work and 22.3% of workers returned partially 3 months after consultation with the OP. Results indicated that workers whose occupational physicians had received the training, RTW self-efficacy increased significantly compared to those whose occupational physicians had participated in the control group. Higher RTW self-efficacy scores were significantly more often associated with full RTW than with no RTW three months later, but the intervention did not affect this association. This study showed that training to enhance guideline adherence of occupational physicians leads to increased RTW self-efficacy in workers short-listed with CMDs during the first months of SA in a real-life occupational health care setting. |
| Van Oostrom *et al.* 2009 | Feasibility Evaluation within a Randomized controlled trial | Baseline and 3 months follow up. | CMDs (stress) | RTW | Workplace intervention (scheduling, job design, communication, training, use of support) | Both employees and supervisors. Employees who had been on sick leave from regular work for 2-8 weeks with distress. | N= 112 (Intervention group= 56, CAU= 56) | Questionnaires | 71.4% in the intervention group. 100% in the Usual care group. | Various | Netherlands | Support from leaders | Participants identified 151 obstacles to RTW relating to job design, communication, mental workload and person-related stress factors. The 281 consensus-based solutions identified were mostly related to job design, communication and training. 725 of these solutions were realized at the evaluation with employee and supervisor. Overall, employees, supervisors and Ops were satisfied with the workplace intervention. Time-investment was the only barrier at implementation reported. |
| Van Oostrom *et al.* 2010 | Randomized controlled trial | 3, 6 & 12 months follow up | CMDs (stress) | Lasting RTW | Stress-related symptoms. | Employees with distress and sick-listed for 2- 8 weeks. Mean age= 48.6 years in intervention group and 49.2 in CAU. Male= 76.7% in intervention group and 80.6% in CAU. | N= 145 (Intervention group= 73, CAU= 72) | Questionnaires, administrative data | 100% in the intervention group, 97.2% in CAU. | Various | Netherlands | Support from leaders, Personal characteristics ( Positive attitude-intention to return) | Overall, the participatory workplace intervention where contacts between employee and supervisors were more intensive and structured, indicated no effects on lasting RTW. However, it significantly reduced time until lasting RTW for employees who at baseline declared intentions to RTW despite symptoms. For employees who showed no baseline intentions to return, intervention did not have any effect. |
| Verbeek *et al.* 2002 | Randomized controlled trial | 3 & 12 months follow up | MSDs (back) | Time until RTW | Time until recurrence, number of days lost, rates of RTW at 3 and 12 months, pain intensity, functional disability and six general health perception scales at 3 and 12 months follow up assessments. | Workers with back pain and on sick leave for less than 1 month. Mean age= 39 years, Male= 33% | N= 120 (intervention group= 61, control group= 59) | Questionnaires | 98% at baseline, 92% after 3 months and 90% after 12 months. | Health care, Education | Netherlands | Support from leaders | There were no significant differences found after 3 and 12 months follow up evaluation in terms of time until return to work or other health outcomes. However, recurrences occurred more frequently in the intervention group compared to the reference group (supervisory support). |
| Vermeulen *et al.* 2011 | Randomized controlled trial | 3, 6, 9 & 12 months | MSDs | Sustainable first RTW | Duration of sickness benefit, pain intensity and functional status. | Unemployed and temporary agency workers sick-listed for 2-8 weeks due to MSDs as main health complaints. Mean age= 44.0 years in the intervention group and 45.6 years in the control group, Male= 57.0% in the intervention group and 63.1% in the control group, Level of education= 57.0% in the intervention group and 60.7% in the control group. | N= 163 (Intervention group= 79, CAU= 84) | Questionnaires | 71.2% at follow up. | Various | Netherlands | Support from leaders | The median duration until sustainable RTW was 161 days in the intervention group compared to 299 days in the usual care group. The new participatory RTW program resulted in a non-significant delay in RTW during the first 90 days, followed by a significant advantage in the RTW rate after 90 days. |
| Volker *et al.* 2015 | Prospective Longitudinal | 2 years follow up | CMDs (anxiety, depression, somatization) | Duration until full RTW. | RTW Self-efficacy. | Sick-listed employees who were currently on sick leave between 4 weeks and 1 years and having access to the internet. Age range= 18-44 years and ages ≥45 years. Female= 51.9% | N= 493 | Questionnaire | 55.6% | Various | Netherlands | Personal characteristics (Self-efficacy, age, gender), Support from leaders and co-workers | RTW self-efficacy was a significant predictor of RTW. In the multivariate model, low RTW self-efficacy, the thought of not being able to work while having symptoms and chronic medical conditions were predictors of a longer duration until RTW. |
| Wahlin *et al.* 2012 | Prospective cohort | 3 month follow up | MSDs, CMDs (stress, depression & adjustment disorder, anxiety, burn-out) | RTW within 3 months | Expectations and self-efficacy, social support, health, functioning and work ability and work conditions. | Being on sick leave for MSDs or mental disorders, age range= 18-65 years, have a good knowledge of Swedish. | N= 699 (MSD group; Clinical group= 314; combined group= 118. Mental disorder group; Clinical group= 146; Combined group= 121) | Questionnaire | 84.1% after 3 months (response to questionnaire) | Various | Sweden | Support from leaders, Personal characteristics (attitude, age, educational level) | Results showed that patients with mental disorders who received the combined intervention (clinical and work-related) returned to work to a higher degree than those who only received clinical intervention. However combined intervention did not affect RTW for patients with MSDs, rather a better work ability and positive expectations of RTW were associated with RTW. The prevalence of work-related interventions was higher for those who were younger and more highly educated. Receiving combined interventions increased the probability of RTW for patients with mental disorders, but not for patients with MSDs. |
| Wainwright *et al.* 2013 | Qualitative | - | MSDs | RTW | Frequent enquiry after health status, Being able to trust employer, Feeling valued, Guidelines about maintaining contact with absent employees and value of the fit note. | Employers who had managed sick leave cases and employees who had experienced sick leave for chronic pain. Be at least 18 years old and able to give informed consent. Be in employment and have needed a sick or fit note within the last year, or be on current sick leave; to have consulted their GP in the last year; to have experience pain lasting over 3 months within the last year. | N= 26 (13 employers and 13 employees) | Semi-structured interviews | 84.6% for employers and 100% for employees. | Charity, Commerce | United Kingdom | Support from leaders | Five themes were elicited. 1. Frequent enquiry after health status was seen as intrusive by some employees but part of good practice by employers and acknowledging this difference was useful. 2. Being able to trust employees due to their performance track record was helpful for employers when dealing with complex chronic pain conditions. 3. Feeling valued increased employee’s motivation to RTW. 4. Guidelines about maintaining contact with absent employees were useful if used flexibly. 5. Both parties valued the fit note for its positive language, interrogative format and biomedical authority. |

# Online Resource 4: Evidence of Sustainable RTW after Ill health (MSDs)


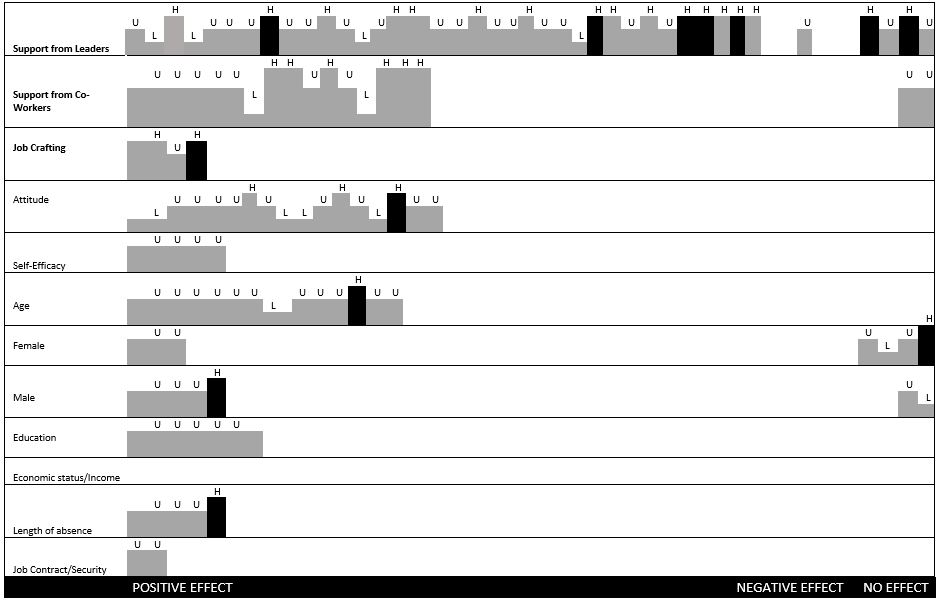


Key: The quality of study is indicated by the height of the bar with a specific designation on it in each row (H to represent very high quality studies, U to represent low quality studies upgraded to high quality based on the GRADE criteria and L to represent low quality studies). Studies with relatively stronger designs (RCT) are indicated with full-tone (black) bars, and weaker study designs (observational and qualitative studies) are indicated with half tone (grey) bars. The harvest plots were combined with a narrative synthesis.

# Online Resource 5: Evidence of Sustainable RTW after Ill health (CMDs)


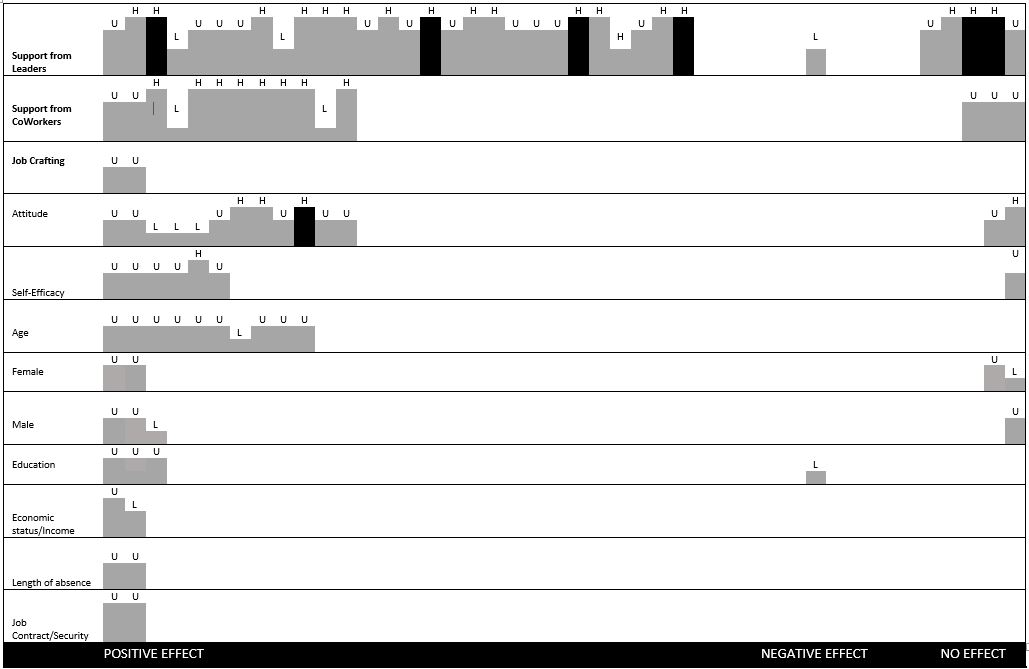


Key: The quality of study is indicated by the height of the bar with a specific designation on it in each row (H to represent very high quality studies, U to represent low quality studies upgraded to high quality based on the GRADE criteria and L to represent low quality studies). Studies with relatively stronger designs (RCT) are indicated with full-tone (black) bars, and weaker study designs (observational and qualitative studies) are indicated with half tone (grey) bars. The harvest plots were combined with a narrative synthesis.

# Online Resource 6: Evidence of Sustainable RTW after Ill health for multiple interaction of factors (MSDs)


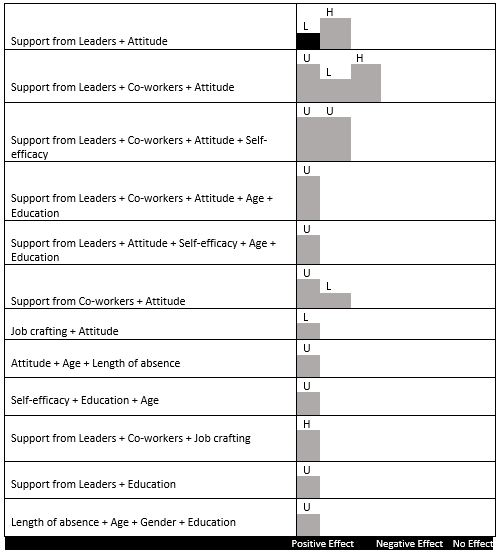


Key: The quality of study is indicated by the height of the bar with a specific designation on it in each row (H to represent very high quality studies, U to represent low quality studies upgraded to high quality based on the GRADE criteria and L to represent low quality studies). Studies with relatively stronger designs (RCT) are indicated with full-tone (black) bars, and weaker study designs (observational and qualitative studies) are indicated with half tone (grey) bars. The harvest plots were combined with a narrative synthesis. (Included studies: Support from leaders + attitude=3, 34; support from leaders + co-workers + attitude= 8, 24; support from leaders + co-workers + attitude + self-efficacy= 10, 11; support from leaders + co-workers+ attitude + age + education= 16; support from leaders + attitude + self-efficacy + age + education= 78; support from co-workers + attitude= 20, 46; job crafting + attitude= 23; attitude + age + length of absence= 32; self-efficacy + education + age= 36; support from leaders, co-workers + job crafting= 38; support from leaders + education= 54; length of absence + age + gender + education= 50)

# Online Resource 7: Evidence of Sustainable RTW after Ill health for multiple interaction of factors (CMDs)


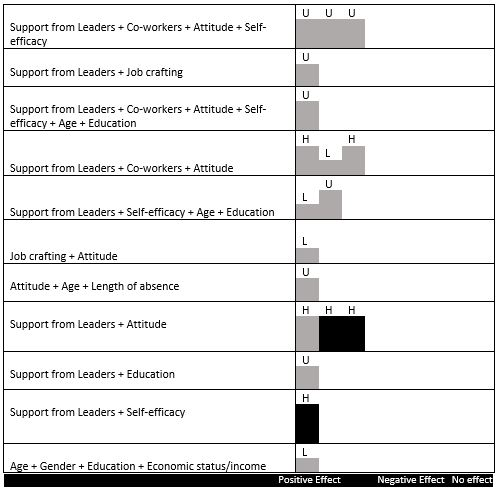


Key: The quality of study is indicated by the height of the bar with a specific designation on it in each row (H to represent very high quality studies, U to represent low quality studies upgraded to high quality based on the GRADE criteria and L to represent low quality studies). Studies with relatively stronger designs (RCT) are indicated with full-tone (black) bars, and weaker study designs (observational and qualitative studies) are indicated with half tone (grey) bars. The harvest plots were combined with a narrative synthesis. (Included studies: Support from leaders + co-workers + attitude+ self-efficacy=10, 11, 77; support from leaders + job crafting= 9; support from leaders + co-workers + attitude + self-efficacy + age + education= 16; support from leaders + co-workers+ attitude= 18, 24, 63; support from leaders + self-efficacy + age + education= 22, 78; job crafting + attitude= 23; attitude + age + length of absence= 32; support from leaders + attitude= 34, 53, 74; support from leaders + education= 54; support from leaders + self-efficacy= 72; age + gender + economic status/income= 62).

# Online Resource 8: Early Intervention Foundation (EIF) Quality Assessment Checklist (RCTs)

| CRITERIA | **3** | **4** | **5** | **13** | **39** | **41** | **49** | **52** | **58** | **60** | **65** | **67** | **68** | **72** | **73** | **74** | **75** | **76** |
| --- | --- | --- | --- | --- | --- | --- | --- | --- | --- | --- | --- | --- | --- | --- | --- | --- | --- | --- |
| Participants completed the same set of measures once shortly before participating in the intervention and once again immediately afterwards | Y | Y | Y | Y | Y | Y | Y | Y | Y | Y | Y | Y | Y | Y | Y | Y | Y | Y |
| Participants were randomly assigned to the treatment and control group through the use of methods appropriate for the circumstances and target population OR sufficiently rigorous quasi- experimental methods (regression discontinuity, propensity score matching) were used to generate an appropriately comparable sample through non-random methods. | Y | Y | Y | Y | Y | N | Y | Y | Y | Y | Y | Y | Y | Y | Y | Y | Y | Y |
| Assignment to the treatment and comparison group was at the appropriate level (e.g., individual, family, school, community). | Y | Y | Y | Y | Y | Y | Y | Y | Y | Y | Y | Y | Y | Y | Y | Y | Y | Y |
| An ‘intent-to-treat’ design was used, meaning that all participants recruited to the intervention participated in the pre/post measurement, regardless of whether or how much of the intervention they received, even if they dropped out of the intervention (this does not include dropping out of the study- which may then be regarded as missing data). | C | Y | Y | Y | Y | Y | Y | Y | Y | Y | Y | Y | Y | Y | Y | Y | Y | Y |
| The treatment and comparison conditions are thoroughly described. | N | Y | Y | Y | Y | Y | Y | Y | Y | Y | Y | Y | Y | Y | Y | Y | Y | Y |
| The extent to which the intervention was delivered with fidelity is clear. | Y | Y | Y | Y | Y | Y | Y | Y | Y | Y | Y | Y | Y | Y | Y | Y | Y | Y |
| The comparison condition provides an appropriate counterfactual to the treatment group. | N | Y | Y | Y | Y | Y | Y | Y | Y | Y | Y | Y | Y | Y | Y | Y | Y | Y |
| The sample is representative of the intervention’s target population in terms of age, demographics and level of need. The sample characteristics are clearly stated. | Y | Y | Y | Y | Y | Y | Y | Y | Y | Y | Y | Y | Y | Y | Y | Y | N | Y |
| The sample is sufficiently large to test for the desired impact. A minimum of 20 participants have completed the measures at both time points within each study group (e.g., a minimum of 20 participants in pre/ post study not involving a comparison group or a minimum of 20 participants in the treatment group AND comparison group). | Y | Y | Y | Y | Y | Y | Y | Y | Y | Y | Y | Y | Y | Y | Y | Y | Y | Y |
| The study has clear processes for determining and reporting drop-out and dose. | Y | Y | Y | Y | Y | C | Y | Y | Y | Y | Y | Y | Y | Y | Y | Y | Y | Y |
| A minimum of 35% of the participants completed pre/ post measures. Overall study attrition is not higher than 65%. | Y | Y | Y | Y | Y | Y | Y | Y | Y | Y | Y | Y | Y | Y | Y | Y | Y | Y |
| There is baseline equivalence between the treatment and comparison group participants on key demographic variables of interest to the study and baseline measures of outcomes (when feasible). | Y | Y | Y | Y | Y | Y | Y | Y | Y | Y | Y | Y | Y | Y | Y | Y | Y | Y |
| Risks for contamination of the comparison group and other confounding factors have been taken into account and controlled for in the analysis (see below) if possible. | N | Y | Y | Y | Y | Y | Y | Y | Y | Y | Y | Y | Y | Y | Y | Y | Y | Y |
| Participants were blind to their assignment to the treatment and comparison group. | N | Y | Y | N | Y | N | Y | C | C | C | N | N | Y | Y | C | N | N | N |
| There was consistent and equivalent measurement of the treatment and control groups at all points when measurement took place. | Y | Y | Y | Y | Y | Y | Y | Y | Y | Y | C | Y | C | Y | Y | Y | Y | Y |
| The study had clear processes for determining and reporting drop-out and dose. Differences between study drop-outs and completers were reported if attrition was greater than 10%. The study assessed and reported on overall and differential attrition. | Y | Y | Y | Y | Y | Y | Y | Y | Y | Y | Y | Y | Y | Y | Y | Y | Y | Y |
| The measures were appropriate for the intervention’s anticipated outcomes and population. | Y | Y | Y | Y | Y | Y | Y | Y | Y | Y | Y | Y | Y | Y | Y | Y | Y | Y |
| The measures used were valid and reliable. This means that the measure was standardised and validated independently of the study and the methods for standardization were published. Administrative data and observational measures may also have been used to measure programme impact, but sufficient information was given to determine their validity for doing this. | Y | Y | Y | Y | Y | Y | Y | Y | Y | Y | Y | Y | Y | Y | Y | Y | Y | Y |
| Measurement was independent of any measures used as part of the treatment. | Y | Y | Y | Y | Y | Y | Y | Y | Y | Y | Y | Y | Y | Y | Y | Y | Y | Y |
| Measurement was blind to group assignment. | Y | Y | Y | Y | Y | Y | Y | Y | Y | Y | Y | Y | Y | Y | Y | Y | Y | Y |
| In addition to any self-reported data (collected through the use of validated instruments), the study also included assessment information independent of the study participants (e.g., an independent observer, administrative data, etc.). | C | Y | Y | Y | Y | Y | Y | Y | Y | Y | Y | Y | Y | Y | Y | Y | Y | Y |
| The methods used to analyse results are appropriate given the data being analysed (categorical, ordinal, ratio/ parametric or non- parametric, etc.) and the purpose of the analysis. | Y | Y | Y | Y | Y | Y | Y | Y | Y | Y | Y | Y | Y | Y | Y | Y | Y | Y |
| Appropriate methods have been used and reported for the treatment of missing data. | C | Y | C | Y | Y | Y | Y | Y | Y | Y | Y | Y | Y | Y | Y | Y | Y | Y |

Online Resource 8: Early Intervention Foundation (EIF) Quality Assessment Checklist (Continued). (Other Quantitative Studies)

| CRITERIA | 1 | 7 | 8 | 9 | 10 | 11 | 12 | 14 | 15 | 16 | 17 | 20 | 21 | 22 | 23 |
| --- | --- | --- | --- | --- | --- | --- | --- | --- | --- | --- | --- | --- | --- | --- | --- |
| Participants completed the same set of measures once shortly before participating in the intervention and once again immediately afterwards | Y | Y | Y | Y | Y | Y | Y | Y | Y | Y | Y | Y | Y | Y | Y |
| Participants were randomly assigned to the treatment and control group through the use of methods appropriate for the circumstances and target population OR sufficiently rigorous quasi- experimental methods (regression discontinuity, propensity score matching) were used to generate an appropriately comparable sample through non-random methods. | - | - | - | Y | - | - | Y | - | Y | - | - | - | Y | - | - |
| Assignment to the treatment and comparison group was at the appropriate level (e.g., individual, family, school, community). | Y | Y | Y | Y | Y | Y | Y | Y | Y | Y | Y | Y | Y | Y | Y |
| An ‘intent-to-treat’ design was used, meaning that all participants recruited to the intervention participated in the pre/post measurement, regardless of whether or how much of the intervention they received, even if they dropped out of the intervention (this does not include dropping out of the study- which may then be regarded as missing data). | - | - | - | N | - | - | - | - | - | - | - | - | - | - | - |
| The treatment and comparison conditions are thoroughly described. | Y | Y | Y | Y | Y | Y | Y | Y | Y | Y | Y | Y | Y | Y | Y |
| The extent to which the intervention was delivered with fidelity is clear. | Y | Y | Y | Y | Y | Y | Y | Y | Y | Y | Y | Y | Y | Y | Y |
| The comparison condition provides an appropriate counterfactual to the treatment group. | Y | Y | Y | Y | Y | Y | Y | Y | Y | C | Y | Y | Y | Y | Y |
| The sample is representative of the intervention’s target population in terms of age, demographics and level of need. The sample characteristics are clearly stated. | Y | Y | Y | Y | Y | Y | Y | Y | Y | Y | Y | Y | Y | N | Y |
| The sample is sufficiently large to test for the desired impact. A minimum of 20 participants have completed the measures at both time points within each study group (e.g., a minimum of 20 participants in pre/ post study not involving a comparison group or a minimum of 20 participants in the treatment group AND comparison group). | Y | Y | Y | Y | Y | Y | Y | Y | Y | Y | Y | Y | Y | Y | Y |
| The study has clear processes for determining and reporting drop-out and dose. | Y | C | Y | Y | Y | Y | Y | Y | Y | Y | Y | Y | Y | Y | Y |
| A minimum of 35% of the participants completed pre/ post measures. Overall study attrition is not higher than 65%. | Y | Y | Y | Y | Y | Y | Y | Y | Y | Y | Y | Y | Y | Y | Y |
| There is baseline equivalence between the treatment and comparison group participants on key demographic variables of interest to the study and baseline measures of outcomes (when feasible). | Y | Y | Y | Y | Y | Y | Y | Y | Y | Y | Y | Y | Y | Y | N |
| Risks for contamination of the comparison group and other confounding factors have been taken into account and controlled for in the analysis (see below) if possible. | Y | C | Y | Y | Y | Y | Y | Y | Y | Y | Y | Y | Y | Y | Y |
| Participants were blind to their assignment to the treatment and comparison group. | - | - | - | - | - | - | - | - | - | - | - | - | - | - | - |
| There was consistent and equivalent measurement of the treatment and control groups at all points when measurement took place. | Y | Y | Y | Y | Y | Y | Y | Y | Y | Y | Y | Y | Y | Y | Y |
| The study had clear processes for determining and reporting drop-out and dose. Differences between study drop-outs and completers were reported if attrition was greater than 10%. The study assessed and reported on overall and differential attrition. | Y | C | Y | Y | Y | Y | Y | Y | Y | C | Y | C | Y | Y | Y |
| The measures were appropriate for the intervention’s anticipated outcomes and population. | Y | Y | Y | Y | Y | Y | Y | Y | Y | Y | Y | Y | Y | Y | Y |
| The measures used were valid and reliable. This means that the measure was standardised and validated independently of the study and the methods for standardization were published. Administrative data and observational measures may also have been used to measure programme impact, but sufficient information was given to determine their validity for doing this. | Y | Y | Y | Y | Y | Y | Y | Y | Y | Y | Y | Y | Y | Y | Y |
| Measurement was independent of any measures used as part of the treatment. | Y | Y | Y | Y | Y | Y | Y | Y | Y | Y | Y | Y | Y | Y | Y |
| Measurement was blind to group assignment. | Y | Y | Y | Y | Y | Y | Y | Y | Y | Y | Y | Y | Y | Y | Y |
| In addition to any self-reported data (collected through the use of validated instruments), the study also included assessment information independent of the study participants (e.g., an independent observer, administrative data, etc.). | Y | Y | Y | Y | Y | Y | Y | Y | Y | N | N | N | Y | Y | Y |
| The methods used to analyse results are appropriate given the data being analysed (categorical, ordinal, ratio/ parametric or non- parametric, etc.) and the purpose of the analysis. | Y | Y | Y | Y | Y | Y | Y | Y | Y | Y | Y | Y | Y | Y | Y |
| Appropriate methods have been used and reported for the treatment of missing data. | Y | Y | Y | Y | Y | Y | Y | Y | Y | Y | Y | Y | Y | Y | Y |

Continued.

| CRITERIA | 24 | 25 | 26 | 28 | 31 | 32 | 33 | 35 | 36 | 37 | 40 | 42 | 43 | 44 | 45 | 46 |
| --- | --- | --- | --- | --- | --- | --- | --- | --- | --- | --- | --- | --- | --- | --- | --- | --- |
| Participants completed the same set of measures once shortly before participating in the intervention and once again immediately afterwards | Y | Y | Y | Y | Y | Y | Y | Y | Y | Y | Y | Y | Y | Y | Y | Y |
| Participants were randomly assigned to the treatment and control group through the use of methods appropriate for the circumstances and target population OR sufficiently rigorous quasi- experimental methods (regression discontinuity, propensity score matching) were used to generate an appropriately comparable sample through non-random methods. | - | - | - | - | - | - | - | Y | - | - | - | - | Y | - | - | - |
| Assignment to the treatment and comparison group was at the appropriate level (e.g., individual, family, school, community). | Y | Y | Y | Y | Y | Y | Y | Y | Y | Y | Y | Y | Y | Y | Y | Y |
| An ‘intent-to-treat’ design was used, meaning that all participants recruited to the intervention participated in the pre/post measurement, regardless of whether or how much of the intervention they received, even if they dropped out of the intervention (this does not include dropping out of the study- which may then be regarded as missing data). | - | - | - | - | - | - | - | - | - | - | - | - | - | - | - | - |
| The treatment and comparison conditions are thoroughly described. | Y | Y | Y | Y | Y | Y | Y | Y | Y | Y | Y | Y | Y | Y | Y | Y |
| The extent to which the intervention was delivered with fidelity is clear. | Y | Y | Y | Y | Y | Y | Y | Y | Y | Y | Y | Y | Y | Y | Y | Y |
| The comparison condition provides an appropriate counterfactual to the treatment group. | Y | Y | Y | Y | Y | Y | Y | Y | Y | Y | Y | Y | Y | Y | Y | Y |
| The sample is representative of the intervention’s target population in terms of age, demographics and level of need. The sample characteristics are clearly stated. | N | Y | Y | Y | Y | Y | Y | Y | Y | Y | Y | Y | Y | Y | Y | N |
| The sample is sufficiently large to test for the desired impact. A minimum of 20 participants have completed the measures at both time points within each study group (e.g., a minimum of 20 participants in pre/ post study not involving a comparison group or a minimum of 20 participants in the treatment group AND comparison group). | Y | Y | Y | Y | Y | Y | Y | Y | Y | Y | Y | Y | Y | Y | Y | Y |
| The study has clear processes for determining and reporting drop-out and dose. | Y | C | Y | Y | Y | Y | Y | Y | Y | Y | Y | Y | Y | Y | Y | N |
| A minimum of 35% of the participants completed pre/ post measures. Overall study attrition is not higher than 65%. | N | Y | Y | Y | Y | Y | Y | Y | Y | Y | Y | Y | Y | Y | Y | N |
| There is baseline equivalence between the treatment and comparison group participants on key demographic variables of interest to the study and baseline measures of outcomes (when feasible). | N | Y | Y | Y | Y | Y | Y | Y | Y | Y | Y | Y | Y | Y | Y | Y |
| Risks for contamination of the comparison group and other confounding factors have been taken into account and controlled for in the analysis (see below) if possible. | Y | Y | Y | Y | Y | Y | Y | Y | Y | Y | Y | Y | Y | Y | Y | Y |
| Participants were blind to their assignment to the treatment and comparison group. | - | - | - | - | - | - | - | - | - | - | - | - | - | - | - | - |
| There was consistent and equivalent measurement of the treatment and control groups at all points when measurement took place. | Y | Y | Y | Y | Y | Y | Y | Y | Y | Y | Y | Y | Y | Y | Y | Y |
| The study had clear processes for determining and reporting drop-out and dose. Differences between study drop-outs and completers were reported if attrition was greater than 10%. The study assessed and reported on overall and differential attrition. | Y | C | Y | Y | Y | Y | Y | Y | Y | Y | Y | Y | Y | Y | Y | N |
| The measures were appropriate for the intervention’s anticipated outcomes and population. | N | Y | Y | Y | Y | Y | Y | Y | Y | Y | Y | Y | Y | Y | Y | Y |
| The measures used were valid and reliable. This means that the measure was standardised and validated independently of the study and the methods for standardization were published. Administrative data and observational measures may also have been used to measure programme impact, but sufficient information was given to determine their validity for doing this. | N | Y | Y | Y | Y | Y | Y | Y | Y | Y | Y | Y | Y | Y | Y | Y |
| Measurement was independent of any measures used as part of the treatment. | Y | Y | Y | Y | Y | Y | Y | Y | Y | Y | Y | Y | Y | Y | Y | Y |
| Measurement was blind to group assignment. | Y | Y | Y | Y | Y | Y | Y | Y | Y | Y | Y | Y | Y | Y | Y | Y |
| In addition to any self-reported data (collected through the use of validated instruments), the study also included assessment information independent of the study participants (e.g., an independent observer, administrative data, etc.). | Y | Y | Y | Y | Y | Y | Y | Y | N | Y | Y | Y | Y | Y | N | Y |
| The methods used to analyse results are appropriate given the data being analysed (categorical, ordinal, ratio/ parametric or non- parametric, etc.) and the purpose of the analysis. | Y | Y | Y | Y | Y | Y | Y | Y | Y | Y | N | Y | Y | Y | Y | Y |
| Appropriate methods have been used and reported for the treatment of missing data. | C | Y | Y | Y | Y | Y | Y | Y | C | C | Y | Y | Y | Y | Y | N |

Continued.

| CRITERIA | 47 | 48 | 50 | 54 | 55 | 57 | 59 | 61 | 62 | 64 | 69 | 70 | 77 | 78 |
| --- | --- | --- | --- | --- | --- | --- | --- | --- | --- | --- | --- | --- | --- | --- |
| Participants completed the same set of measures once shortly before participating in the intervention and once again immediately afterwards | Y | Y | Y | Y | Y | Y | Y | Y | Y | Y | Y | Y | Y | Y |
| Participants were randomly assigned to the treatment and control group through the use of methods appropriate for the circumstances and target population OR sufficiently rigorous quasi- experimental methods (regression discontinuity, propensity score matching) were used to generate an appropriately comparable sample through non-random methods. | - | - | - | - | - | - | - | - | - | N | Y | N | - | - |
| Assignment to the treatment and comparison group was at the appropriate level (e.g., individual, family, school, community). | Y | Y | Y | Y | Y | Y | Y | Y | Y | Y | Y | Y | Y | Y |
| An ‘intent-to-treat’ design was used, meaning that all participants recruited to the intervention participated in the pre/post measurement, regardless of whether or how much of the intervention they received, even if they dropped out of the intervention (this does not include dropping out of the study- which may then be regarded as missing data). | - | - | Y | - | - | - | - | - | - | N | - | - | - | Y |
| The treatment and comparison conditions are thoroughly described. | Y | Y | Y | Y | Y | Y | Y | Y | Y | Y | Y | Y | Y | Y |
| The extent to which the intervention was delivered with fidelity is clear. | Y | Y | Y | Y | Y | Y | Y | Y | Y | Y | Y | Y | Y | Y |
| The comparison condition provides an appropriate counterfactual to the treatment group. | Y | Y | Y | Y | Y | Y | Y | Y | Y | N | Y | Y | Y | Y |
| The sample is representative of the intervention’s target population in terms of age, demographics and level of need. The sample characteristics are clearly stated. | Y | Y | Y | Y | Y | Y | Y | Y | Y | Y | Y | Y | Y | Y |
| The sample is sufficiently large to test for the desired impact. A minimum of 20 participants have completed the measures at both time points within each study group (e.g., a minimum of 20 participants in pre/ post study not involving a comparison group or a minimum of 20 participants in the treatment group AND comparison group). | Y | Y | Y | Y | Y | Y | Y | Y | Y | Y | Y | Y | Y | Y |
| The study has clear processes for determining and reporting drop-out and dose. | Y | Y | Y | C | Y | Y | Y | Y | Y | Y | Y | Y | Y | Y |
| A minimum of 35% of the participants completed pre/ post measures. Overall study attrition is not higher than 65%. | Y | Y | Y | Y | Y | Y | N | Y | Y | Y | Y | Y | Y | Y |
| There is baseline equivalence between the treatment and comparison group participants on key demographic variables of interest to the study and baseline measures of outcomes (when feasible). | Y | Y | Y | Y | Y | Y | Y | Y | Y | Y | Y | Y | Y | Y |
| Risks for contamination of the comparison group and other confounding factors have been taken into account and controlled for in the analysis (see below) if possible. | Y | Y | Y | Y | Y | Y | Y | Y | N | Y | Y | Y | Y | Y |
| Participants were blind to their assignment to the treatment and comparison group. | - | - | - | - | - | - | - | - | - | N | - | - | - | - |
| There was consistent and equivalent measurement of the treatment and control groups at all points when measurement took place. | Y | Y | Y | Y | Y | Y | Y | Y | Y | Y | Y | Y | Y | Y |
| The study had clear processes for determining and reporting drop-out and dose. Differences between study drop-outs and completers were reported if attrition was greater than 10%. The study assessed and reported on overall and differential attrition. | Y | Y | Y | C | Y | Y | Y | Y | Y | Y | Y | Y | Y | Y |
| The measures were appropriate for the intervention’s anticipated outcomes and population. | Y | Y | Y | Y | Y | Y | Y | Y | Y | Y | Y | Y | Y | Y |
| The measures used were valid and reliable. This means that the measure was standardised and validated independently of the study and the methods for standardization were published. Administrative data and observational measures may also have been used to measure programme impact, but sufficient information was given to determine their validity for doing this. | Y | Y | Y | Y | Y | Y | Y | Y | Y | Y | Y | Y | Y | Y |
| Measurement was independent of any measures used as part of the treatment. | Y | Y | Y | Y | Y | Y | Y | Y | Y | Y | Y | Y | Y | Y |
| Measurement was blind to group assignment. | Y | Y | Y | Y | Y | Y | Y | Y | Y | Y | Y | Y | Y | Y |
| In addition to any self-reported data (collected through the use of validated instruments), the study also included assessment information independent of the study participants (e.g., an independent observer, administrative data, etc.). | Y | Y | Y | N | Y | Y | Y | Y | Y | N | Y | Y | Y | Y |
| The methods used to analyse results are appropriate given the data being analysed (categorical, ordinal, ratio/ parametric or non- parametric, etc.) and the purpose of the analysis. | Y | Y | Y | Y | Y | Y | Y | Y | Y | Y | Y | C | Y | Y |
| Appropriate methods have been used and reported for the treatment of missing data. | Y | Y | Y | C | Y | Y | Y | Y | Y | N | Y | Y | Y | Y |

# Online Resource 9: CASP Checklist for Qualitative and Mixed studies

| **Study inclusion checklist (screening questions)** | 2 | 6 | 18 | 19 | 27 | 29 | 30 | 34 | 38 | 51 | 53 | 56 | 63 | 66 | 71 | 79 |
| --- | --- | --- | --- | --- | --- | --- | --- | --- | --- | --- | --- | --- | --- | --- | --- | --- |
| **1. Is a qualitative methodology appropriate?** | Y | Y | Y | Y | Y | Y | Y | Y | Y | Y | Y | Y | Y | Y | Y | Y |
| **Consider;**  Does the research seek to interpret or illuminate the actions and/or subjective experiences of research participants?  Is qualitative research the right methodology for addressing the research goal? | Y | Y | Y | Y | Y | Y | Y | Y | Y | Y | Y | Y | Y | Y | Y | Y |
| **2. Is the research design appropriate for addressing the aims of the research?**  **Consider;**  Has the researcher justified the research design (e.g. have they discussed how they decided which method to use)? | Y | Y | Y | Y | Y | Y | Y | Y | Y | Y | Y | Y | Y | Y | Y | Y |
| 1. **Is there a clear statement of findings?**   **Consider;**  Are the findings made explicit?  Is there adequate discussion of the evidence both for and against the researcher’s arguments?  Has the researcher discussed the credibility of their findings (e.g. triangulation, respondent validation, more than one analyst)?  Are the findings discussed in relation to the original research question? | Y | Y | Y | Y | Y | Y | Y | Y | Y | Y | Y | Y | Y | Y | Y | Y |
| **The following criteria should be considered for each study to be included in the review (i.e. those for which the answers to all the questions were “yes”).** |  |  |  |  |  |  |  |  |  |  |  |  |  |  |  |  |
| 1. **Was the data collected in a way that addressed the research issue?**   **Consider:**  Is the setting for data collection justified?  Is it clear what methods were used to collect data? (E.g. focus group, semi-structured interview etc.)? Has the researcher justified the methods chosen?  Has the researcher made the process of data collection explicit (e.g. for interview method, is there an indication of how interviews were conducted, or did they use a topic guide)?  If methods were modified during the study, has the researcher explained how and why? Is the form of data clear (e.g. tape recordings, video material, notes etc.)? | Y | Y | Y | Y | Y | Y | Y | Y | Y | Y | Y | Y | Y | Y | Y | Y |
| 1. **Was the recruitment strategy appropriate to the aims of the research?**   **Consider:**  Has the researcher explained how the participants were selected?  Have they explained why the participants they selected were the most appropriate to provide access to the type of knowledge sought by the study?  Is there are any discussion around recruitment and potential bias (e.g. why some people chose not to take part)? Is the selection of cases/ sampling strategy theoretically justified? | Y | Y | Y | Y | Y | Y | Y | Y | Y | Y | Y | Y | Y | Y | Y | Y |
| **6. Was the data analysis sufficiently rigorous?**  **Consider:**  If there is an in-depth description of the analysis process?  If thematic analysis is used, is it clear how the categories/themes were derived from the data?  Does the researcher explain how the data presented were selected from the original sample to demonstrate the analysis process?  Are sufficient data presented to support the findings?  Were the findings grounded in/ supported by the data?  Was there good breadth and/or depth achieved in the findings?  To what extent are contradictory data taken into account?  Are the data appropriately referenced (i.e. attributions to (anonymised) respondents)? | Y | Y | Y | C | Y | Y | Y | Y | Y | Y | Y | Y | Y | N | Y | Y |
| **7. Has the relationship between researcher and participants been adequately considered?**  **Consider:**  Has the researcher critically examined their own role, potential bias and influence during (a) formulation of the research questions (b) data collection, including sample recruitment and choice of location?  How has the researcher responded to events during the study and have they considered the implications of any changes in the research design? | Y | Y | Y | Y | Y | Y | Y | Y | Y | Y | Y | Y | Y | N | Y | Y |
| **8. Have ethical issues been taken into consideration?**  **Consider:**  Are there sufficient details of how the research was explained to participants for the reader to assess whether ethical standards were maintained?  Has the researcher discussed issues raised by the study (e.g. issues around informed consent or confidentiality or how they have handled the effects of the study on the participants during and after the study)?  Have they adequately discussed issues like informed consent and procedures in place to protect anonymity? Have the consequences of the research been considered i.e. raising expectations, changing behaviour?  Has approval been sought from an ethics committee? | Y | Y | Y | Y | Y | Y | Y | Y | Y | Y | Y | Y | Y | Y | Y | Y |
| **9.** **Contribution of the research to wellbeing impact questions?**  **Consider:**  Does the study make a contribution to existing knowledge or understanding of what works for wellbeing? E.g. are the findings considered in relation to current practice or policy? | Y | Y | Y | Y | Y | Y | Y | Y | Y | Y | Y | Y | Y | Y | Y | Y |

# Online Resource 10: List of included studies

| Author | Year | Title |
| --- | --- | --- |
| Ahltrom *et al*. | 2013 | Workplace rehabilitation and supportive conditions at work; a prospective study |
| Andersen *et al.* | 2014 | How do workers with common mental disorders experience a multidisciplinary return to work intervention? A qualitative study. |
| Anema *et al*. | 2003 | Participatory ergonomics as a return to work intervention: a future challenge |
| Arends *et al.* | 2013 | Prevention of recurrent sickness absence in workers with common mental disorders: results of a cluster-randomised controlled trial. |
| Arnetz *et al.* | 2003 | Early workplace intervention for employees with musculoskeletal related absenteeism; a prospective controlled intervention study |
| Baril *et al.* | 2003 | Management of return to work programs for workers with musculoskeletal disorders; a qualitative study in three Canadian provinces. |
| Bernacki *et al.* | 2000 | A facilitated early return to work program at a large urban medical center. |
| Besen *et al.* | 2015 | Returning to work following low back pain; towards a model of individual psychosocial factors. |
| Bond and Bunce. | 2001 | Job control mediates change in a work re-organization intervention for stress reduction. |
| Brouwer *et al.* | 2009 | Behavioural determinants as predictors of return to work after long-term sickness absence; an application of the theory of planned behaviour |
| Brouwer *et al.* | 2010 | A prospective study of return to work across health conditions; perceived work attitude, self-efficacy and perceived social support. |
| Brouwer *et al.* | 2011 | Return to work self-efficacy; development and validation of a scale in claimants with musculoskeletal disorders |
| Bűltmann *et al.* | 2009 | Coordinated and tailored work rehabilitation (CTWR): A randomized controlled trial with economic evaluation undertaken with workers on sick leave due to musculoskeletal disorders. |
| Burtler *et al.* | 2007 | It pays to be nice; employer-worker relationships and the management of back pain claims |
| Crook and Moldofsky | 1994 | The probability of recovery and return to work from work disability as a function of time. |
| D’Amato and Zijlstra | 2010 | Toward a climate for work resumption; the nonmedical determinants of return to work. |
| De Rijk *et al.* | 2008 | Gender differences in return to work patterns among sickness absentees and their associations with health; a prospective cohort study in the Netherlands. |
| De Vries *et al.* | 2014 | Perceived impeding factors for return to work after long-term sickness absence due to major depressive disorder: A concept mapping approach |
| Dionne *et al.* | 2013 | Obstacles to and facilitators of return to work after work-disabling back pain; The worker’s perspective |
| Dunstan *et al.* | 2013 | What leads to the expectation to return to work? Insights from a theory of planned behaviour (TPB) model of future work outcomes |
| Durand *et al.* | 2000 | Therapeutic return to work: Rehabilitation in the workplace. |
| Ekberg *et al.* | 2015 | Early and late return to work after sick leave; predictors in a cohort of sick-listed individuals with common mental disorders |
| Ekbladh | 2010 | Return to work; The predictive value of the worker role interview (WRI) over two years |
| Ekbladh *et al.* | 2004 | The worker role interview- preliminary data on the predictive validity of return to work of clients after an insurance medicine investigation |
| Engstrom and Janson | 2007 | Stress-related absence and return to labour market in Sweden |
| Franche *et al.* | 2007 | The impact of early workplace-based return to work strategies on work absence duration; a 6-month longitudinal study following an occupational musculoskeletal injury |
| Friesen *et al.* | 2001 | Return to work; the importance of human interactions and organizational structures. |
| Gallagher *et al.* | 1989 | Determinants of return-to-work among low back pain patients |
| Hatchard *et al.* | 2012 | Workers’ perspective on self-directing mainstream return to work following acute mental illness: Reflections on partnerships |
| Haugli *et al.* | 2011 | What facilitates return to work? Patients experiences 3 years after occupational rehabilitation |
| Haveraaen *et al.* (2016) | 2016 | Do psychological job demands, decision control and social support predict return to work three months after return to work (RTW) programme? The rapid-RTW cohort study |
| Heijbel *et al.* | 2006 | Return to work expectation predicts work in chronic musculoskeletal and behavioural health disorders; Prospective study with clinical implications |
| Heijbel *et al.* | 2013 | Implementation of a rehabilitation model for employees on long-term sick leave in the public sector; Difficulties, counter-measures and outcomes |
| Hoefsmit *et al.* | 2014 | Environmental and personal factors that support early return to work; A qualitative study using the ICF as a framework |
| Hu *et al.* | 2014 | Predictors of return to work and duration of absence following work-related hand injury |
| Huijs *et al.* | 2012 | Differences in predictors of return to work among long term sick listed employees |
| Janssen *et al.* | 2003 | The demand-control-support model as a predictor of return to work |
| Jakobsen and Lillefejell | 2014 | Factors promoting a successful return to work; from an employer and employee perspective |
| Jensen *et al.* | 2012 | Sustainability of return to work in sick-listed employees with low-back pain. Two-year follow-up in a randomized clinical trial comparing multidisciplinary and brief intervention. |
| Johansson *et al.* | 2006 | Return to work and adjustment latitude among employees on long-term sickness absence |
| Karlson *et al.* | 2010 | Return to work after a workplace-oriented intervention for patients on sick-leave for burnout; A prospective study |
| Karlson *et al.* | 2014 | Long-term stability of return to work after a workplace-oriented intervention for patients on sick leave for burnout |
| Krause *et al.* | 2001 | Psychosocial job factors and return-to-work after compensated low back injury: A disability phase-specific analysis |
| Labriola *et al.* | 2006 | Multilevel analysis of individual and contextual factors as predictors of return to work |
| Lagerveld *et al.* | 2010 | Return to work among employees with mental health problems; development and validation of a self-efficacy questionnaire |
| Laisne *et al.* | 2013 | Biopsychosocial determinants of work outcomes of workers with occupational injuries receiving compensation; A prospective study |
| Lammerts *et al.* | 2016 | Longitudinal associations between biopsychosocial factors and sustainable return to work of sick-listed workers with a depressive or anxiety disorder |
| Lederer *et al.* | 2012 | Gender differences in personal and work-related determinants of return to work following long-term disability: A 5year cohort study. |
| Loisel *et al.* | 1997 | A population-based, randomized clinical trial on back pain management. |
| Lydell *et al.* | 2009 | Predictive factors of sustained return to work for persons with musculoskeletal disorders who participated in rehabilitation |
| Lysaght and Larmour-Trode | 2008 | An exploration of social support as a factor in the return to work process |
| Marhold *et al.* | 2001 | A cognitive behavioural return to work program: effects on pain patients with a history of long-term versus short-term sick leave. |
| Martin *et al.* | 2015 | Barriers and facilitators for implementation of a return-to-work intervention for sickness absence beneficiaries with mental health problems: results from three Danish municipalities. |
| Muijzer *et al.* | 2011 | Influence of efforts of employer and employee on return to work process and outcomes |
| Nielsen *et al.* | 2010 | Predictors of return to work in employees sick-listed with mental problems: findings from a longitudinal study |
| Nielsen *et al.* | 2013 | Encounters between workers sick-listed with common mental disorders and return to work stakeholders. Does workers’ gender matter? |
| Nieuwenhuijsen *et al.* | 2004 | Supervisory behaviour as a predictor of return to work in employees absent from work due to mental health problems. |
| Opsahl *et al.* | 2016 | Do expectancies of return to work and job satisfaction predict actual return to work in workers with long lasting LBP? |
| Post *et al.* | 2005 | Work-related determinants of return to work off employees on long-term sickness absence |
| Poulsen *et al.* | 2014 | Effect of the Danish return-to-work program on long-term sickness absence: results from a randomized controlled trial in three municipalities. |
| Reiso *et al.* | 2003 | Back to work: Predictors of Return to Work among patients with Back disorders certified as sick. |
| Roelen *et al.* | 2012 | Employees sick-listed with mental disorders; Who returns to work and when? |
| Selander *et al.* | 2015 | Contact with the workplace during long-term sickness absence and worker expectations of return to work |
| Shaw *et al.* | 2008 | An investigation of a workplace-based return to work program for shoulder injuries |
| Shiri *et al.* | 2011 | The effect of workplace intervention on pain and sickness absence caused by upper-extremity musculoskeletal disorders |
| Stahl and Stiwne | 2014 | Narratives of sick leave, return to work and job mobility for people with common mental disorders in Sweden |
| Steenstra *et al.* | 2006 | Economic evaluation of multi-stage return to work program for workers on sick-leave due to low back pain. |
| Steenstra *et al.* | 2009 | What works best for whom? An exploratory, subgroup analysis in a randomized, controlled trial on the effectiveness of a workplace intervention in low back pain patients on return to work. |
| Stoltenberg & Skov | 2010 | Determinants of return to work after long-term sickness absence in six Danish Municipalities. |
| Tenhiala *et al.* | 2013 | Organizational justice, sickness absence and employee age. |
| Tjulin *et al.* | 2011 | The social interaction of return to work explored from co-workers experiences. |
| Van Beurden *et al.* | 2015 | Effect of an intervention to enhance guideline adherence of occupational physicians on return to work self-efficacy in workers sick-listed with common mental disorders |
| Van Oostrom *et al.* | 2009 | A participatory workplace intervention for employees with distress and lost time; A feasibility evaluation within a randomized controlled trial |
| Van Oostrom *et al.* | 2010 | A workplace intervention for sick-listed employees with distress; results of a randomized controlled trial |
| Verbeek *et al.* | 2002 | Early occupational health management of patients with back pain. |
| Vermeulen *et al.* | 2011 | A participatory return to work intervention for temporary agency workers and unemployed workers sick-listed due to musculoskeletal disorders; Results of a randomized controlled trial |
| Volker *et al.* | 2015 | Return to work self-efficacy and actual return to work among long-term sick-listed employees |
| Wahlin *et al.* | 2012 | Association between clinical and work-related interventions and return to work for patients with musculoskeletal or mental disorders |
| Wainwright *et al.* | 2013 | Return to work with chronic pain: employer’s and employee’s views |
